# The Epigenetic Landscape and Exposome of Non-Melanoma Skin Cancer: Mechanisms, Biomarkers, and Therapeutic Perspectives

**DOI:** 10.3390/genes17040477

**Published:** 2026-04-17

**Authors:** Adrian Albulescu, Alina Fudulu, Iulia Virginia Constantin (Iancu), Adriana Plesa, Irina Huica, Anca Botezatu

**Affiliations:** 1 Molecular Virology Department, Stefan S. Nicolau Institute of Virology, Romanian Academy, 030304 Bucharest, Romania; adrian.albulescu@virology.ro (A.A.); iulia.iancu@virology.ro (I.V.C.); adriana.plesa@virology.ro (A.P.); irina.huica@virology.ro (I.H.); anca.botezatu@virology.ro (A.B.); 2 Pharmacology Department, National Institute for Chemical Pharmaceutical Research and Development, 031299 Bucharest, Romania

**Keywords:** non-melanoma skin cancer, epigenetics, exposome

## Abstract

Accounting for over 1.2 million new diagnoses worldwide in 2022, non-melanoma skin cancer (NMSC) represents the most common human cancer, predominantly manifesting as basal cell carcinoma (BCC) and squamous cell carcinoma (SCC). NMSC serves as a powerful natural model for studying how environmental exposure, the exposome, reprograms the epigenome to drive carcinogenesis. Chronic ultraviolet radiation (UVR), the dominant risk factor, induces DNA damage and inflammation that dysregulate epigenetic enzymes (e.g., DNMTs, HDACs). These effects are layered with perturbations from β-HPV infection and cutaneous dysbiosis, altering DNA methylation, histone modifications, and non-coding RNA and miRNA expression in a multistep carcinogenic process. This review synthesizes the central role of epigenetic regulation as the critical interface between genetic susceptibility and cumulative exposome factors in NMSC pathogenesis. We integrate how UVR, HPV, and inflammation converge to remodel the keratinocyte epigenome. Finally, we evaluate the translational potential of this knowledge for refined risk stratification through epigenetic biomarkers and discuss emerging therapeutic strategies, including epidrugs, that target these dysregulated pathways for advanced NMSC management.

## 1. Introduction

Skin cancer is the most common human malignancy type worldwide and is classified as melanoma and non-melanoma skin cancer (NMSC). The term keratinocyte carcinoma (KC) has been increasingly adopted to emphasize the common cellular origin of these tumors, though “NMSC” remains the prevailing clinical nomenclature. NMSC is one of the most common malignancies in fair-skinned populations worldwide [1]. According to GLOBOCAN, the incidence of NMSC was estimated at around 1,235,000 new cases in 2022, worldwide, ranking fifth of all cancers in both sexes and all ages [2]. The most common types of NMSC are basal cell carcinoma (BCC) and squamous cell carcinoma (SCC), along with other malignant tumors of the skin such as cutaneous lymphomas, Kaposi’s sarcoma, and angiosarcoma [3]. Whilst sporadic BCC develops de novo and accounts for 80% of all NMSC, SCC arises from precursor lesions of actinic keratosis (AK) and Bowen’s disease and represents a multistep accumulation of genetic damage [4].

Besides its high prevalence, NMSC represents a unique model for studying environment–epigenome interactions, as keratinocytes are continuously exposed to potent epi-mutagens such as ultraviolet radiation (UVR), cutaneous HPV infection, pollutants, and inflammatory stimuli. These exposures reshape DNA methylation patterns, chromatin structure, and non-coding RNA networks, making epigenetic deregulation a central contributor to NMSC initiation and progression.

Actinic keratosis (AK) was described as an early carcinoma in situ due to dysplastic keratinocytes similar to SCC [5]. NMSC generally occurs in adults after the age of 40 years, and men have a higher incidence of BCC compared to women. While most other malignancies have had stable or declining incidence rates, the NMSC incidence is increasing with a younger patient age at diagnosis, with UV radiation accounting for almost 90% of the cases [6]. Patients with BCC are at 10 times the risk of developing a further BCC in comparison to the general population [7]. Although BCC metastases are extremely rare, they are highly destructive to surrounding tissues if left untreated. SCCs are malignant epidermal keratinocyte neoplasms and manifest in most cases from pre-existing skin malignancies. SCC presents as a rough keratosis papule protuberance and is characterized by rapid growth. Biological and epidemiological studies have assessed the population attributable factor (PAF) range for SCC at approximately 0.5–0.7 in lightly pigmented populations and around 0.5–0.9 for BCC in terms of UVR exposure. For a skin tone between light and deep, a 0.1 multiplier was used to derive incidence estimates, and for darker shades, a multiplier of 0.018 was applied. This method refers to the proportion of the total burden of disease that is due to exposure to an environmental risk factor, in this case UV radiation [8]. Despite this increased risk, no long-term follow-up is required for BCC once the primary tumor has been cured. A total of 95% of recurrences for SCC occur within 5 years, with 70–80% of these recurrences occurring within the first 2 years. The NCCN recommends 3–6 monthly follow-up for 2 years and then 6–12 months for a further 3 years [9]. Sentinel lymph node biopsy has been reported to identify early microscopic lymph node metastasis but is yet to show survival advantage, and further studies in this area are required [10,11].

Even if BCC does not metastasize and SCC rarely metastasizes, there are exceptions represented by immunosuppressed patients, e.g., in solid organ transplant recipients or patients with AIDS. The most frequent neoplasms in solid organ transplant recipients are SCC (with a 65-fold increase in incidence), BCC (with a 10-fold increase in incidence), Kaposi’s sarcoma (KS), melanoma and Merkel cell carcinoma, in order [12,13].

NMSC is one of the most powerful natural systems for examining how environmental exposures reprogram the epigenome. Keratinocytes experience chronic UVR-induced DNA lesions, oxidative stress, and inflammation, all of which activate or inhibit epigenetic enzymes such as DNMTs, TETs, HDACs, and HATs. In addition, commensal or persistent β-HPV infections and cutaneous dysbiosis alter chromatin accessibility and non-coding RNA expression, providing layered epigenetic perturbations rarely observed in internal malignancies. In this context, given the central role of epigenetic alterations in environmentally driven carcinogenesis, this review synthesizes current knowledge on epigenetic mechanisms underlying NMSC, including DNA methylation, histone modifications, chromatin remodeling, and non-coding RNA regulation. We further integrate how UVR, HPV, inflammation, and other exposome factors reprogram the epigenome and evaluate potential epigenetic biomarkers and therapeutic targets relevant to keratinocyte carcinoma.

## 2. Genetic Factors Involved in NMSC Development

Multiple factors are involved in NMSC development, e.g., sun exposure, artificial UVR (tanning beds and lamps), aging, diet, smoking, treatment modalities (radiotherapy, phototherapy, psoralen, long-wave ultraviolet radiation (PUVA), immunosuppressant drugs (cyclosporin A, methotrexate)), and work-related exposures such as arsenic, tar products, and chemical carcinogens (petroleum refining, pesticide manufacturing, etc.) [14,15,16,17].

One of the most important mutated genes related to UVR exposure is the well-known tumor suppressor p53 [18,19,20]. Mutations in p53 have been reported to be characteristic of AK and SCC, being identified in 69% to over 90% of invasive SCC. Other mutated genes found in SCC include *WNT*, *Ras*, *p16INK4*, *NF-κB* and *c-Myc* [14,21,22]. Regarding the UVR types, it was observed that UVA radiation is involved in skin stem cell transformation, while UVB radiation leads to inflammatory responses and oncogenesis [18].

Moreover, genetic predisposition to cutaneous squamous cell carcinoma (SCC) is well established in several hereditary cancer syndromes. Individuals with inherited defects in DNA repair and genomic stability, such as mutations in nucleotide excision repair genes, telomere maintenance genes and mismatch repair genes, face significantly increased SCC risk [23]. A recent systematic review by Binstock et al. shows strong associations between SNPs in pigmentation genes and cSCC risk, independent of skin phenotype. *MC1R* red-hair variants (Arg151Cys, Arg160Trp) and the *ASIP* haplotype showed the highest correlations, suggesting additional roles in immune and UV-response pathways. SNPs in DNA repair genes have also been implicated in both SCC and BCC, with variants in thymine DNA glycosylase, *ERCC8*, *ERCC3*, *PALB2*, *DMC1*, *MGMT*, *CHEK2*, and *MSH6* linked to increased risk (Table 1) [24].

## 3. Epigenetic Factors Involved in NMSC Development

Development of cancer depends on significant alterations in the tumor microenvironment (TME), where surrounding cells undergo epigenetic reprogramming in response to tumor-secreted factors. As noted by Hanahan et al., signaling molecules and growth factors from the tumor can modify the behavior of nearby stromal cells, disrupting their normal regulatory pathways [29]. External factors, such as ultraviolet radiation (UVR), further contribute to epigenetic dysregulation, influencing chromatin structure and DNA damage response.

Studies suggest that heterochromatin, due to its dense and peripheral nuclear positioning, may absorb UVB-induced damage, acting as a protective barrier for transcriptionally active euchromatin [30]. This selective shielding could explain variations in mutation rates across genomic regions. Additionally, DNA methylation plays a critical role in cancer by silencing tumor suppressor genes through promoter hypermethylation [31]. Epigenetic defects—rather than genetic mutations alone—can impair key cellular processes, including DNA repair and cell cycle control [32].

### 3.1. DNA Methylation in NMSC

DNA methylation alterations represent a well-established hallmark of cancer [33]. Given that aging is a major risk factor for tumorigenesis, the shared epigenetic features between aged and cancerous cells—including lamina-associated domain hypomethylation, focal CpG-island promoter hypermethylation, and epigenetic shift—are not surprising [34].

Compared to other malignancies, DNA methylation patterns in non-melanoma skin cancer (NMSC) remain understudied, with most research focusing on CpG-island hypermethylation of individual genes. Key examples include tumor suppressor genes involved in DNA repair (e.g., *MLH1*, *MGMT*, *BRCA1*), cell cycle regulation, apoptosis, and signal transduction [35]. These alterations, often arising early in tumorigenesis, can be reversed by demethylating agents and hold promise as diagnostic, prognostic, and therapeutic biomarkers.

In basal cell carcinoma (BCC), hypermethylation-associated silencing affects genes such as *CDH13*, *SFN*, *TNFRSF10C*, *FHIT*, *SHH*, *APC*, *SFRP5*, and *RASSF1* [36,37,38,39,40]. Also, an aberrant methylation pattern was shown for *FHIT* [33], *BCL7a*, *PTPRG*, *TP73*, *p14ARF* (encoding tumor suppressor p14) and *FAS* promoter in [32]. Methylation in the *PTCH* gene was shown to likely play a minor role in carcinogenesis [41].

Similarly, cutaneous squamous cell carcinoma (cSCC) exhibits inactivation of tumor suppressors (*CDKN2A*, *RB1*, *CDH1*) [42,43], while *MIR204* silencing has been implicated in the progression from actinic keratosis (AK) to invasive cSCC [44].

### 3.2. Non-Coding RNAs in NMSC

Beyond methylation, non-coding RNAs (ncRNAs), such as microRNAs (miRNAs) and long non-coding RNAs (lncRNAs), are increasingly recognized as regulators of oncogenes and tumor suppressors in skin cancer. These molecules modulate gene expression and contribute to malignant progression, highlighting the complexity of epigenetic influences in carcinogenesis.

#### 3.2.1. MicroRNAs

MicroRNAs (miRNAs) are short non-coding RNAs (between 17 to 25 nucleotides) that regulate gene expression post-transcriptionally. The mechanisms underlying their dysregulation in cancer are complex and likely multifactorial, involving altered miRNA expression, transcription factor activity, and miRNA sequence mutations, reflecting the intricate regulatory networks in human metabolism [45]. In cutaneous SCC, distinct miRNAs are dysregulated by UVA/UVB radiation and associated with malignancy compared to healthy skin. These miRNAs exert oncogenic or tumor-suppressive effects by targeting key genes (*PTEN*, *TP53*, *VEGFA*, *MMP13*, and *LZTS1*), contributing to carcinogenesis through synergistic mechanisms [46]. Compared to BCC, SCC shows higher rates of regional metastases, which often correlate with poorer prognosis [47,48].

The most significantly upregulated and downregulated miRNAs in SCC and BCC are summarized in Table 2 and Table 3.

#### 3.2.2. Long Non-Coding RNAs in NMSC

Over the past decade, long non-coding RNAs (lncRNAs) have emerged as critical regulators of organism development and disease pathogenesis, including carcinogenesis [69]. While the mutational landscape of cutaneous squamous cell carcinoma (cSCC) and basal cell carcinoma (BCC) is well characterized, the involvement of lncRNAs in the signaling networks underlying skin malignancies remains largely unexplored [32]. To date, only a limited number of lncRNAs have been implicated in cSCC progression, including the oncogenic *LINC00162* (*PICSAR*) and *HOTAIR* as well as the tumor suppressor *LINC00520* [59,70,71].

Moreover, Mancini et al. identified a significant downregulation of the lncRNA uc.291 in cSCC, being associated with elevated *ACTL6A* expression. This uc.291 suppression enhances ACTL6A-mediated inhibition of the SWI/SNF complex near epidermal differentiation complex (EDC) promoters, leading to reduced expression of loricrin and *LEC1C*. Mechanistically, *BRG1* (SWI/SNF subunit) and uc.291 jointly regulate the dedifferentiation phenotype in aggressive cSCCs. While BCCs showed similar mRNA trends, protein-level discrepancies imply additional suppression mechanisms. Public datasets corroborated uc.291 and BRG1/BRM downregulation as conserved SCC traits, revealing a novel epigenetic tumor suppressor pathway in SCC [72].

Also, another study demonstrates that the long non-coding RNA *CCAT2* is significantly overexpressed in non-melanoma skin cancers (NMSCs) and correlates with disease progression. These results suggest *CCAT2* as a promising biomarker for NMSC diagnosis and prognostic evaluation [73].

A study comparing tumor tissues from cSCC and BCC to healthy marginal skin revealed significant upregulation of *BBOX1-AS1*, *HOXB7*, and *IGF2BP1*, with cSCC exhibiting markedly higher expression levels than BCC. These biomarkers were correlated with tumor grade and lesion burden, suggesting a potential role in skin cancer pathogenesis [74].

## 4. HPV Role and Mechanism of Non-Melanoma Skin Cancer Oncogenesis

Cutaneous human papillomaviruses (HPVs) have been implicated in a variety of skin diseases. Large-scale metagenomic analyses, including whole-genome shotgun sequencing of over 100 healthy volunteers, detected HPV in 69% of skin samples—the highest prevalence among tested body sites (vagina: 42%; mouth: 30%; gut: 17%) [75]. Notably, >95% of viral sequences in skin were papillomaviruses, predominantly beta and gamma genera [76]. Frequent co-infections with multiple HPV types suggest potential competitive or synergistic interactions that may influence immune evasion and viral persistence.

Cutaneous HPV infections are highly prevalent in infants and young children, demonstrating early viral exposure [77]. Common types (e.g., HPV2, 7, 27, 57 [alpha]; HPV23, 75 [beta]; HPV4, 65 [gamma]; HPV1 [mu]) typically cause benign warts (verrucae vulgaris, plantaris, plana) that often regress spontaneously [78]. However, immunocompromised individuals, particularly organ transplant recipients (OTRs), exhibit higher prevalence (48–92% within 5 years post-transplant) and severe manifestations (e.g., confluent warts) [79].

The HIM study revealed that beta/gamma HPV infections persist for 6–11 months, with older individuals showing higher susceptibility [80]. Notably, viral DNA detection alone cannot confirm active infection or immune exposure; serological studies are essential to assess seroconversion and immune dynamics [79], particularly in preclinical models.

The role of human papilloma virus (HPV) in NMSC development is contradictory despite all the studies realized in this field, but growing epidemiological and mechanistic evidence indicates that certain commensal HPVs serve as key factors in skin carcinogenesis—particularly for SCC [81].

While Ally et al. showed that the presence of HPV DNA in tissue lesions and antibody seropositivity to cutaneous HPV, especially genus-beta, were associated with SCC, no significant association between HPV genus-beta presence and BCC was found [82]. Also, two other studies could not bring any proof of HPV genus-alpha involvement in BCC development [83,84]. On the other hand, it was shown that the presence of HPV was associated with SCC in immunosuppressed individuals, suggesting an etiological role for this virus in SCC, maybe because of the inability of this group to have a proper immune response [85]. Iannacone et al. reported the association between BCC and HPV in a cohort study. Antibodies were found for all five HPV genera (alpha, beta, gamma, mu, nu). Moreover, the authors detected the presence of viral DNA in lesional tissue from of beta types 5, 8, 9, 12, 14, 15, 17, 19, 20, 21, 22, 23, 24, 25, 36, 37, 38, 47, 49, 75, 76, 80, 92, 93 and 96, gamma types 4, 65, 95, 60, 48, 50, 88, 101, 103, 108, 109, 112, 116, 119, 121, 123, alpha types 2, 3, 10, 27, 57, and mu type 1. The study showed also that beta-HPV 5 and 8 are high-risk types for SCC, but no HPV types seem to be characteristic for BCC [86].

The role of HPVs in NMSC development seems to be linked to UV radiation exposure [87]. It was shown that cutaneous HPV types are frequently found in sites extensively exposed to sun, and several studies regarding E6 and E7 viral proteins of genus-beta HPV types can interfere with the mechanisms of DNA repair after UVR exposure, inactivate p53, and lead to immortalization. Regarding BCC, it was not demonstrated whether HPV presence could have a role in dysregulation of the Hedgehog pathway, with the possibility of cutaneous HPV [82].

Mucosal HPVs primarily target p53 and pRB, driving cellular transformation and unchecked proliferation by disrupting cell cycle arrest, apoptosis, metabolism, and immune signaling. In contrast, cutaneous papillomaviruses employ distinct mechanisms: their E6 proteins (e.g., HPV1, HPV8) bind *MAML1* to inhibit NOTCH signaling—a strategy shared by animal PVs (BPV1, MmuPV1, MnPV). *NOTCH*, activated by MAML1-p300-CREBBP complexes, normally suppresses tumors and promotes keratinocyte differentiation; its frequent mutation in cSCCs or HPV-mediated disruption sustains proliferative states. HPV5/8 further impair differentiation via E6-directed p300 degradation, reducing keratin 1/10 and involucrin expression [81].

## 5. Exposome

**Figure 1:**
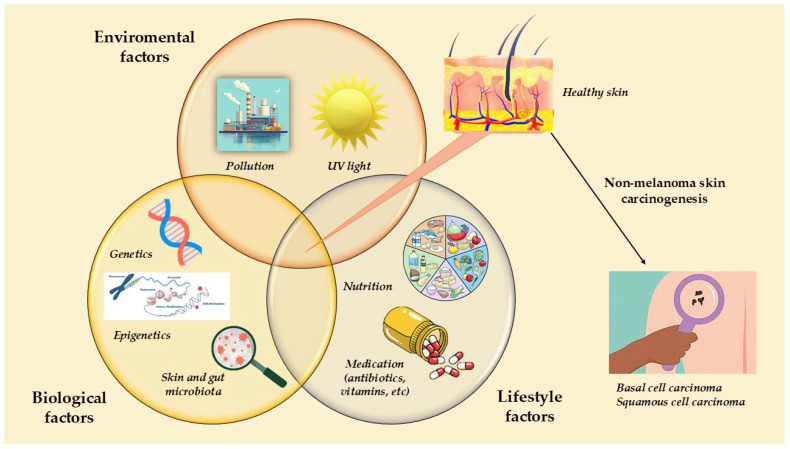
The exposome–epigenome interface in NMSC pathogenesis.

The exposome, comprising all lifelong environmental exposures such as ultraviolet radiation, pollution, diet, and skin microbiota, converges on shared epigenetic mechanisms that drive NMSC pathogenesis. These exposures induce epigenetic alterations in keratinocytes, including changes in DNA methylation, histone modifications, and chromatin remodeling, which collectively regulate gene expression and chromatin accessibility. Dysregulation of these processes promotes abnormal keratinocyte proliferation and impaired differentiation, facilitating the progression from healthy skin to cutaneous squamous cell carcinoma or basal cell carcinoma, the two major forms of non-melanoma skin cancer. A schematic overview of these mechanisms and their contribution to NMSC development is presented in Figure 1.

### 5.1. Environmental Factors

UV radiation has been classified by the International Agency for Research on Cancer (IARC) as a Group 1 carcinogen [88]. Its contribution to skin cancer development varies according to both the duration and intensity of exposure: long-term, cumulative exposure is primarily linked to squamous cell carcinoma (SCC), whereas short-term, intense sun exposure is a major cause of melanoma and basal cell carcinoma (BCC) [89]. BCC was associated with intermittent and childhood sun exposure, while SCC was correlated with chronic UV exposure [90].

Previous studies have demonstrated a causal relationship between UV radiation and skin conditions such as BCC, SCC, and actinic keratosis (AK) [88]. Evidence consistently indicates that UV radiation is a strong risk factor for NMSC, particularly among individuals with lighter skin phototypes.

Furthermore, the World Health Organization (WHO) and the International Labour Organization (ILO) have recognized occupational exposure to UV radiation as a risk factor for NMSC [91]. This causal link has been documented among outdoor workers, including agricultural and construction workers, who spend prolonged periods outdoors during peak sunlight hours.

The mechanism underlying the effect of UV light involves inducing direct DNA mutation via covalent bonding between adjacent pyrimidines primarily producing cyclobutane pyrimidine dimers and promoting chromatin condensation, leading to increased apoptosis (UVB) and formation of reactive oxygen species (UVA) [92].

It was also shown that the use of tanning devices and phototherapy for various skin disease treatments, especially associated with psoralen (UVR), increases the risk for both cancer types [17,18]. UVR therefore induces gene mutations, immunosuppression, oxidative stress and inflammatory responses.

Inflammation was also proposed as an UVR-exposure-derived mechanism that leads to skin carcinogenesis. Chronic inflammation by infiltrating immune cells contributes to skin tumor progression [6,93]. Free radicals produced by UVR exposure leads to indirect DNA damage and induces immunosuppression. UVR immunosuppression is mediated by Langerhans cells (LCs), which lead to the loss of the dendritic network, and then the LCs migrate to lymph nodes. Moreover, migrated LCs activate natural killer T cells (NK-T cells) that produce interleukin IL-4 and induce Treg to secrete IL-10; both interleukins show immunosuppressive activity [94,95].

A recent longitudinal study showed that the other link between UV irradiation and skin inflammation is represented by vitamin D receptor (VDR) gene polymorphisms that can favor NMSC development. The authors showed that patients with rs2228570, rs927650 and rs1544410 dominant or rs7975232 and rs739837 recessive genotypes were associated with a lower risk of developing BCC [96].

The central mechanism of NMSC development is the inflammatory process, which triggers tumor growth. In order to develop new therapeutic approaches, a better understanding of the inflammation pathways is necessary, as this could unlock potential treatment targets in pathologies associated with skin inflammation. The main specific actors, such as IL-17 and IL-22, were found to be secreted by infiltrated T lymphocytes in both BCC and SCC cell lines, and proliferation and migration abilities were significantly increased by in vitro IL-17 and IL-22 [6].

### 5.2. Pollution

Another factor potentially contributing to skin cancer is the extensive use of pesticides. Pesticides can enter the human body through multiple routes, with the skin being the most exposed organ during field spraying activities. In occupational settings, the incidence of skin cancer is significantly higher among farmers using pesticides [97]. Exposure to certain pesticide components, such as arsenic and polychlorinated biphenyls (PCBs), has shown the strongest carcinogenic potential for skin cancer [98] Moreover, cumulative evidence indicates that climate-related environmental changes—such as global atmospheric temperature increases, ozone layer deterioration, and air contamination—are likely fueling the global rise in skin cancer incidence, with long-term implications for public health [99].

### 5.3. Microbiota, Skin Virome

Microorganisms are estimated to contribute to approximately 20% of all tumors worldwide [100]. Advances in contemporary sequencing technologies have generated high-throughput data on the microbiome from various tissues, revealing novel pathogens enriched in multiple cancer types compared to either peri-tumoral tissues or healthy controls. Microbiome dysbiosis has been shown to promote oncogenesis [101].

The skin microbiota plays a multifaceted role, including the induction of immune tolerance in early life, production of anti-microbial agents and immune-regulatory metabolites, promotion of wound healing, and enhancement of barrier functions. Certain components of the skin microbiota have been shown to inhibit tumor progression, whereas dysbiosis may compromise the microbial community’s protective function [102].

Importantly, UV exposure can alter the skin microbiota, triggering the excessive generation of reactive oxygen species (ROS), apoptosis, and inflammation [103]. Evidence suggests that skin microbiota can influence tumor development. Certain skin bacteria produce cis-urocanic acid, a compound that modulates UV-induced immune suppression and melanoma progression [104].

*Staphylococcus aureus* has been frequently associated with the oncogenesis process [105]. The detection of *S. aureus* in biopsies and swab samples from SCC cases has demonstrated a strong correlation with this type of skin cancer [106]. Furthermore, another study reported that in SCC-lesional skin, *S. aureus* represented the most abundant bacterial species [107]. Madhusudhan et al. (2020) suggested that the high abundance of *S. aureus* in SCC may influence *human β-defensin-2* expression, thereby promoting SCC progression [108].

In actinic keratosis and SCC lesions, a reduced abundance of bacteria from the genus *Propionibacterium* has been observed [109]. Propionibacterium produces coproporphyrin III, a compound that promotes *S. aureus* aggregation. A decreased presence of *Propionibacterium acnes* and *Propionibacterium granulosum* has been linked to increased susceptibility to skin cancers [110].

Also, in a study by Voigt et al., the authors characterized the skin microbiome in SCC patients and its precursor, AK, compared with healthy skin from patients and controls. Using whole-genome shotgun sequencing, they identified disease-associated microbial shifts, including a marked decrease in the commensal *Cutibacterium acnes* and an increase in the pathobiont *S. aureus* in AK and SCC lesions. These findings provide a high-resolution baseline for microbial associations with SCC, supporting further research into their mechanistic role in carcinogenesis [109].

Moreover, microbial metabolites, particularly short-chain fatty acids (SCFAs) like butyrate and propionate produced by gut microbiota, can influence host gene expression by serving as donors for epigenetic modifications [111]. These SCFAs regulate epigenetic processes primarily by inhibiting histone deacetylase (HDAC) activity, thereby increasing histone acetylation and altering genome-wide modification patterns [112]. While SCFAs are primarily produced by anaerobic microbes in the gut, certain skin-colonizing bacteria such as *C. acnes* and *Staphylococcus epidermidis* also possess this capacity. In vitro studies demonstrate that SCFAs from *C. acnes* can inhibit HDAC 8 and 9 activity, thereby influencing TLR-mediated immune and inflammatory responses in keratinocytes and sebocytes [113]. Consequently, shifts in the composition of the skin microbiota may serve as a significant epigenetic factor by altering the local metabolomic environment.

Recent research showed that certain commensal skin bacteria, such as a specific strain of *S. epidermidis* and *C. acnes*, influence the cutaneous molecular environment through immune activation and barrier regulation. These microbes trigger pro-inflammatory cytokine production via NF-κB, MAPK, and inflammasome pathways while also modulating miRNA expression—particularly miR-146 through TLR2 signaling in keratinocytes and sebocytes—which in turn negatively regulates immune responses [114].

Additionally, *S. epidermidis* may protect against non-melanoma skin cancer by producing 6-N-hydroxyaminopurine (6-HAP), an anti-proliferative compound that targets neoplastic cells. This finding has important implications for prevention and treatment in high-risk individuals [115].

The convergence of microbiome research and epigenetic therapy opens new avenues for exploiting SCFAs as a new class of epidrugs targeting the skin microenvironment and NMSC management.

### 5.4. Medication (Immunosuppression, Certain Medications)

A higher likelihood of developing NMSC has been linked to the usage of immunosuppressive medications such as those prescribed following organ transplantation or for the treatment of HIV infection and other immune disorders, with the risk being influenced by multiple factors, such as the type of drug, its dosage, the length of treatment, and the patient’s individual characteristics [116].

There are reports suggesting that certain drugs, especially those used to treat chronic conditions like hypertension or cholesterol-lowering drugs (statins), may be associated with an elevated risk of non-melanoma skin cancer [117,118].

Medication-induced photosensitivity represents an important modifiable component of the skin exposome. Hydrochlorothiazide (HCTZ), a common antihypertensive, exhibits well-documented photosensitizing properties associated with cSCC risk. Epidemiological studies demonstrate a clear dose–response relationship, with long-term HCTZ use increasing cSCC risk by approximately 3- to 4-fold [119]. Mechanistically, HCTZ absorbs UVA radiation, triggering p53 activation, DNA damage, and pro-inflammatory responses in exposed skin [120]. These findings highlight the need for regular skin surveillance in patients on long-term HCTZ therapy and consideration of alternative antihypertensives in high-risk individuals.

### 5.5. Nutrition and Vitamins

Regarding the potential therapeutic role of vitamins in NMSC, the data are far from conclusive.

Although the antioxidant role of vitamins C and E is quite well documented, they were reported to act more in prevention rather than in therapy [121]. Vitamin A derivatives (retinoids) are used in both topical and systemic administration in various skin conditions, including cancers. The drugs in this class have different toxicity and biological activity [122]. Several studies report the use of nicotinamide (an amide derivative of vitamin B3) as a protective factor against NMSC and a possible therapy [123,124]. This molecule acts by promoting genomic stability and induces the repairing of UV-damaged DNA [125]. The authors of a case–control study observed that less NMSCs were developed in patients under nicotinamide treatment than in controls [126]. On the other hand, very poor associations were made between the oral intake of niacin (B3) vitamin and reduced risk of skin cancers, and even a slightly increased risk of BCC was reported associated with niacin intake [127].

A series of food-derived supplements have been shown to have beneficial effects on health and have been intensely studied in cancer prevention or treatment.

Turmeric plays an important role in the emergence of skin cancers due to the UVR induced generation of such molecular species along with chronic inflammation. Turmeric’s anti-inflammatory properties have been postulated to be caused by its ability to *inhibit NF-κB*, *TNF-α*, *cyclooxygenase-2* (*COX-2*), *Cyclin D1*, *c-myc*, *B-cell lymphoma-2* (*Bcl-2*), *inducible nitric oxide synthase* (*iNOS*) and interleukins. In skin cancers, the NF-κB pathway has been shown to be upregulated. Another anti-carcinogenic effect of curcumin on skin cancer was associated with inhibition of AKT/mTOR and ERK signaling [128,129,130,131].

Ginger is known to inhibit tumor growth and angiogenesis in human ovarian cancer cells and has been proven to exhibit anti-inflammatory and anti-angiogenic effects by downregulating *NF-κB*, *IL-8* and *VEGF* expression.

Garlic has been shown to inhibit TNF-α, IL-6 expression and anti-inflammatory cytokine levels, and a correlation has been found between dose and skin tumor incidence in vivo. Its anti-cancer properties are thought to be caused by diallyl disulfide (DADS) upregulation of antioxidant enzymes such as SOD, catalase, heme oxygenase (HO), GPx and the nuclear accumulation of nuclear factor-like 2 (Nrf2) [132,133].

Cloves have been shown to inhibit the activation of NF-κB and the expression of LPS-stimulated cytokines. Cloves have also been shown to be strong ROS scavengers and to inhibit the formation of malondialdehyde (MDA). Another effect displayed by cloves is the downregulation of *Bcl-2*, *COX-2*, *c-myc* and *H-ras* [134,135].

In rosemary, carnosol and ursolic acid are responsible for 90% of its antioxidant activity and have a strong inhibitory effect on TPA-induced skin carcinogenesis. Carnosol has ROS chelator activity, inhibits 5-lipoxygenase activity, and suppresses matrix metallopeptidase 9 mRNA by downregulating *NF- κB*, *c-jun*, *AKT*, *p38*, *JNK* and *ERK1/2*. Rosemary extract has been shown to have protective effects against skin tumorigenesis, and the mechanism through which it acts is thought to involve the elevation of GSH levels.

Saffron’s anti-tumor properties are caused by its ability to inhibit the activation of inflammatory cytokines, PI3K/AKT, Wnt signaling and transcription factors such as AP-1. Oral infusion in mice has shown that it leads to higher levels of antioxidants such as glutathione S-transferase (GST), catalase, superoxide dismutase (SOD) and glutathione peroxidase (GPx) [136,137,138,139].

Capsaicin has been shown to inhibit the NF-κB pathway along with *p65* [140]. Many studies have underlined the anti-inflammatory, anti-proliferation, anti-microbial, antioxidant and anti-tumor properties of spices, and although a cure-all option is not available, the use of spices to aid in the prevention/treatment of cancer is a safe bet [141].

While many spices are well-documented for their ability to suppress NF-κB activation—a master regulator of pro-inflammatory gene expression—it is crucial to recognize that the organism receives epigenetic anti-inflammatory signals from both dietary constituents and microbial metabolism [142]. Dietary phytochemicals, including polyphenols found in turmeric (curcumin), ginger (gingerols), and other plant-based foods, function as natural “epigenetic modifiers” by regulating gene expression through DNA methylation, histone modifications, and microRNA expression [143]. For instance, these compounds can demethylate promoter regions of antioxidant genes like *NRF2* and inhibit inflammatory responses via hypermethylation of inflammation-related genes [144]. Concurrently, the gut microbiota generates metabolites—particularly short-chain fatty acids (SCFAs) such as butyrate and propionate—that serve as essential substrates and cofactors for epigenetic enzymes, influencing histone acetylation and DNA methylation patterns [145]. Key metabolic intermediates, including acetyl-CoA, SAM, and α-KG, act as “metabolic signaling molecules” that couple cellular energy status with epigenetic regulation. Moreover, specific microbial metabolites like phloroglucinol have been shown to induce long-lasting innate immune training in hematopoietic progenitors through the aryl hydrocarbon receptor (AhR), conferring sustained anti-inflammatory protection [146]. This intricate interplay between diet-derived phytochemicals and microbiota-derived metabolites underscores a dual pathway through which environmental factors shape epigenetic landscapes and immune homeostasis [147].

Exposure to carcinogenic chemicals, especially arsenic, determines the increasing expression of several proteins, including keratin 7 and keratin 9. It has also been reported that topical application of 12-O-tetradecanoylphorbol-13-acetate (TPA) in C57BL/6-resistant and DBA/2-sensitive mouse models determined increased expression of S100, A8 and A9 proteins involved in inflammation that affect skin neoplastic growth [148,149].

Hence, in a prospective study reported in 2017, QSkin, involving over 40,000 patients, it was shown that for smokers, the risk for developing SCC was high without a link to the duration and/or intensity of smoking [150].

## 6. Biomarkers—Predisposition, Diagnostic and Prognostic

Biomarkers serve as essential molecular tools for stratifying patients across the disease continuum, from risk assessment to clinical outcome. This chapter categorizes biomarkers according to their four primary roles: predisposition, diagnostic, prognostic and predictive biomarkers.

Predisposition biomarkers are defined by heritable genetic alterations that confer increased disease susceptibility. In the context of cutaneous malignancies, germline mutations in genes involved in DNA repair (*XPA-G*, *FANC*), telomere maintenance (*DKC1*, *TERT*), tumor suppression (*TP53*, *PTEN*), and hedgehog signaling (*PTCH1*) underpin well-characterized cancer predisposition syndromes, including xeroderma pigmentosum, dyskeratosis congenita, and Gorlin syndrome (Table 1) [25,151]. These inherited defects establish a baseline vulnerability upon which environmental factors, such as ultraviolet radiation, converge to drive carcinogenesis (Table 4) [151].

In basal cell carcinoma (BCC), hypermethylation-associated silencing affects genes such as CDH13, SFN, TNFRSF10C, FHIT, SHH, APC, SFRP5, and RASSF1 [36,37,38,39,40]. Similarly, cutaneous squamous cell carcinoma (cSCC) exhibits inactivation of tumor suppressors (CDKN2A, RB1, CDH1) (Table 5) [42,43,44].

## 7. Old, New and Future Therapies

The gold standard of treatment for NMSCs is surgical resection with histological control of margins. It is known that more than 95% of NMSC tumors can be controlled surgically, being curable. Most common techniques are tangential shave removal, curettage, electrodessication, Mohs micrographic surgery (MMS), and standard surgical excision [190,191]. The main issue arises when these are not viable options and an alternative is needed.

Immunotherapy is an important option when surgical excision is not possible. In addition, systemic and topical pharmacotherapy, cryotherapy (CT), photodynamic therapy (PDT), laser, and radiotherapy (RT) are also used [191,192].

The common feature of all the above-mentioned treatments is that they are quite unspecific without targeting the tumor itself or its environment. In addition, they have high rates of treatment failure, morbidity, and mortality; hence alternative treatment modalities for patients with aggressive or advanced disease are needed.

One of the most frequent alternative treatments is represented by topical pharmacotherapy. Factors such as multifocal or multiple tumors, indistinct lesion boundaries, localization in cosmetically sensitive or difficult-to-treat areas, and a history of hypertrophic scars and keloid favor the use of this type of approach [193]. There are several topical agents that are currently used in the treatment of NMSC, some of which are approved by the FDA. Furthermore, they are easy to apply and well tolerated. Because BCC represents 75% of all skin cancers, a better understanding of its pathogenesis paralleled the development of immunotherapy, which may represent a promising alternative in BCC treatment [194].

Imiquimod is the most attractive immunomodulator for topical treatment of both benign and malignant skin states due to its potent antiviral and anti-tumoral effects. It acts as a toll-like receptor 7 (TLR-7) agonist in the tumor cells and promotes interferon-alpha (IFN-α), tumor necrosis factor-alpha (TNF-α) and other cytokines (IL-1, IL-12, IL-6, IL-8, and IL-10) to increase T helper 1-type immunity (TH-1), and it was first approved for the treatment of actinic keratosis (AK) and superficial BCC [195].

SCC carcinogenesis is initiated and promoted by UV light, which leads to different gene mutations such as p53 and EGFR. EGFR mutation causes downstream signaling, which leads to cell cycle progression, reduced apoptotic capacity, and angiogenesis, and metastatic phenotype EGFR inhibitors are considered to suppress EGFR ligand (TGF-α and EGF)-binding activity. The most described EGFR competitive inhibitors are cetuximab and panitumumab, which are monoclonal antibodies [196].

Cetuximab was approved for head and neck SCC (HNSCC) treatment but not for cutaneous SCC. There is a prospective trial (phase II study) that used cetuximab as a first-line single-drug therapy in patients with unresectable squamous cell carcinoma of the skin (SCCS), and it demonstrated efficiency when considered as a therapeutic choice, particularly for elderly patients in whom chemotherapy is not applicable anymore [197].

Another convincing documentation indicates that BCC etiology is highly dependent on the abnormal activation of Hedgehog signaling pathway [198,199], which also plays an important role in SCC and MM [200,201]. In a selection of sporadic BCCs [202], activating somatic mutations were found in smoothened protein (SMO) and patched genes (PTCH), which are the most frequently mutated genes [203]. For this reason, patients with basal cell carcinoma can be treated by blocking the Hedgehog pathway [204].

Two noteworthy selective inhibitors of the SMO receptor are vismodegib and sonidegib. Vismodegib inhibits the Hh pathway and restores the pathway blockade by binding to the SMO extracellular domain [205]. Both inhibitors (vismodegib (Erivedge^®^) and sonidegib (Odomzo^®^)) were approved the FDA and EMA, with vismodegib being first [206,207,208].

In high-risk BCC cases, when the surgical excision margins are positive after intervention and/or negative margins are not feasible with MMS, both inhibitors are usually recommended for treatment by the National Comprehensive Cancer Network (NCCN) [209].

Hh pathway inhibitors were detected in achieved-resistance mechanisms, and in some measure, they were responsible for a short response term [210]. A trial conducted with patients resistant to vismodegib also indicated resistance after administration of sonidegib, pointing to probable cross-resistance [211].

Over the last few years, research has led to the conclusion that NMSC is strongly linked to immune status, as shown by the large number of cases in organ transplant patients who are undergoing continuous immunosuppression [212]. The current landscape of oncology is being changed by immunotherapy, which is based on the interaction between the immune system and antigens exposed on the surface of cancer cells [194]. This brings in increased interest in immunomodulators, or upregulators of the immune response, in the treatment of various forms of NMSC. An increased number of approaches designed to enhance the host’s immune response against cancer cells and/or cancer cell antigenicity have been assessed. Thus, when patients are poor surgical candidates and/or apply for noninvasive therapy, immunomodulators are becoming a fundamental strategy in NMSC treatment [213].

Immunotherapy includes a large diversity of concepts and methods that are classified in older immunotherapies that lead to nonspecific activation of immune cells that react against tumors (i.e., immunostimulatory cytokines such as IL-2 and IFN) and recent immunotherapies that lead to a specific immune response (i.e., immune checkpoint inhibitors) [194]. Older immunotherapies, such as interferon (IFN) administration, gained attention in SCC treatment following evidence (presence) of HPV in this type of lesion and its antiviral activity. Trials of intralesional IFNs were conducted to assess their efficiency and safety in BCC treatment, and IFN-α-2b was successfully evaluated for SCC and keratoacanthoma treatment [214]. Efficacy rates for intralesional IFN-α seem to be high, ranging from 67 to 86% [215]. A 22% global complete response was assessed by intralesional IFN-γ. Surprisingly, another effective strategy is represented by a combination of topical imiquimod and IFN-α-2b in BCC treatment [216].

This new era of immunotherapy, based on the checkpoint blockade, offers more hope due to encouraging data in head and neck cancer from therapies with anti-PD-1 antibodies and the presence of a high UV-mutation burden in CSCC. It was observed that CSCC development in the epidermis is stimulated by overexpression of PD-1 ligands. The evidence was validated through clinical programs that led to FDA approval of an anti-PD1 antibody developed by Regeneron and Sanofi, known as Cemiplimab [206]. Cemiplimab generates a ~50% response in patients with advanced cutaneous squamous cell carcinoma, being associated with adverse events that are similar to those seen with other PD-1 inhibitors [217].

Patients with no prior systemic treatment and a median age of 80 years having unresectable CSCC received an anti-PD1 antibody, pembrolizumab, that showed a response rate of 42% and a median progression-free survival of around 7 months [218].

Is it known that combined therapy is frequently associated with development of immune-related adverse events (irAEs) and acute reactions such as cytokine release syndrome. This type of effect may result from intravenous immunotherapeutic methods. Therefore, localized or tumor-directed immunotherapy could be a way to reduce the incidence and severity of irAEs, allowing for the use of combination therapies for a synergistic effect.

While conventional therapies—including surgery, immunotherapy, and targeted agents—remain the mainstay of NMSC treatment, a growing body of evidence highlights the potential of epigenetic-based approaches as a novel therapeutic avenue. Epidrugs, which include DNA methyltransferase (DNMT) inhibitors, histone deacetylase (HDAC) inhibitors, bromodomain and extraterminal (BET) inhibitors, and EZH2 inhibitors, are designed to reverse aberrant epigenetic modifications that drive tumorigenesis [219,220]. In cutaneous malignancies, epigenetic dysregulation—such as promoter hypermethylation of tumor suppressor genes and altered histone acetylation patterns—contributes to disease progression and therapeutic resistance. DNMT inhibitors (e.g., 5-azacytidine and decitabine), already used in hematological malignancies, have shown promising results in preclinical solid tumor models, including melanoma, by reactivating silenced tumor suppressor genes and modulating the tumor microenvironment. Similarly, HDAC inhibitors (e.g., vorinostat, romidepsin) can restore normal acetylation patterns, thereby influencing transcription, DNA repair, and apoptotic pathways [221]. Emerging preclinical evidence also supports the combination of epigenetic modifiers with immune checkpoint inhibitors to overcome immunotherapy resistance, as epigenetic alterations can impair tumor neoantigen presentation and modify the immune microenvironment [222]. Although most clinical data to date derive from melanoma studies, the underlying epigenetic mechanisms are equally relevant to NMSC pathogenesis, warranting further investigation into epidrug-based strategies for squamous cell carcinoma and basal cell carcinoma.

Finally, the epigenetic regulatory functions of SCFAs, particularly their ability to inhibit HDAC activity and influence gene expression, open new avenues for epidrug-based therapies in NMSC. Given that dysbiosis-associated loss of SCFA-producing bacteria such as *C. acnes* characterizes NMSC pathogenesis, restoring SCFA levels, either through bacterial recolonization or direct metabolite supplementation, represents a conceptually novel approach. Such strategies could re-establish normal epigenetic control mechanisms and modulate the inflammatory microenvironment, positioning SCFAs as key players in next-generation NMSC therapeutics [223,224].

Thus, integrating epidrugs into future therapeutic regimens—either as single agents or in combination with existing immunotherapies—represents a promising direction for improving outcomes in high-risk or treatment-refractory NMSC patients.

## 8. Conclusions

NMSC, specifically basal cell carcinoma (BCC) and squamous cell carcinoma (SCC), serve as a powerful model for studying environment–epigenome interactions. Unlike internal malignancies, keratinocytes are continuously exposed to external “epi-mutagens” such as ultraviolet radiation (UVR), pollutants, and viral infections. Epigenetic deregulation is a central contributor to the initiation and progression of NMSC. This involves a layered system of perturbations, including DNA methylation (silencing tumor suppressors), histone modifications, and the activity of non-coding RNAs (miRNAs and lncRNAs), that modulate gene expression.

## Figures and Tables

**Table 1:** Genetic changes and polymorphisms (SNPs) associated with NMSC.

	Gene/Proteins Biomarkers		Functions/Role	Ref.
Genetic changes, polymorphisms (SNPs)	SCC and BCC	*MC1R* (melanocortin 1 receptor) (Arg151Cys, Arg160Trp)	Control of melanogenesis	[25]
		*ERCC8*, *ERCC3* (ERCC excision repair)	Nucleotide excision repair	[25,26]
		*PALB2* (partner and localizer of BRCA2), *DMC1* (DNA meiotic recombinase 1)	Homologous recombinational repair	[25,26]
		*MGMT* (O-6-methylguanine-DNA methyltransferase)	Direct reversal repair	[25,26]
		*CHEK2* (checkpoint kinase 2)	DNA damage signal transduction	[25]
		*MSH6* (mutS homolog 6)	Mismatch repair	[25,26]
		mtDNA4977 deletion	Mitochondrial oxidative energy metabolism	[27,28]

**Table 2:** Oncogenic microRNAs in BCC and SCC carcinogenesis.

	miRNA	Associated Functions	Ref.
miRNAs with increased expression levels in BCC	*miRNA-21*	Inhibits multiple tumor suppressors, including *PTEN* and *PDCD4*	[49]
	*miRNA-146a*	Regulates inflammatory immune responses by coordinating myeloid and lymphocyte activity, thereby influencing both innate and adaptive immunity	[50]
	*miRNA197-5p*	Possible involvement in the metastatic process	[51]
	*miRNA-425-5p* *miRNA-433*	Progression of premalignant lesions to malignancy	[52]
	*miRNA-17*	Regulated in vitro through MAPK/ERK-mediated phosphorylation of *TRBP*	[53,54]
	*miRNA-18a* *miRNA-18b*	Cell growth and the inhibition of programmed cell death	[53,54]
	*miRNA-19b*	Promotes increased cell proliferation and inhibits apoptosis	[53,54]
	*miRNA-125a-5p*	Apoptosis inducer	[53,54]
	*miRNA-182*	Inhibits *FOXO1*	[53,54]
	*miRNA-148a* *miRNA-143* *miRNA-378*	-	[49]
	*miRNA-455-3p* *miRNA-455-5p* *miRNA-542-5p*	-	[53,54]
miRNAs with increased expression levels in SCC	*miRNA-21*	Key to skin SCC growth and persistence	[55]
	*miRNA-221*	Significantly increases cell proliferation	[56]
	*miRNA-135b*	Enhances keratinocyte migration and tumor invasiveness in early SCC	[57]
	*miRNA-365*	Through *NFIB* suppression, reduces *CDK4/CDK6* levels.	[58]
	*miRNA-31*	Downregulates the tumor suppressor *RhoBTB1* in A-431 cSCC cells, promoting proliferation and invasion	[59]
	*miRNA-18a*	Associated with both Sonic Hedgehog signaling and cSCC development	[59]
	*miRNA-424*	Modulates angiogenesis by regulating cell-autonomous angiogenic functions	[59]
	*miRNA-130b*	Inhibits *TP53INP1*, a critical p53-mediated anti-cancer protein	[59]
	*miRNA-374a* *miRNA-196a* *miRNA-455-5p* *miRNA-766* *miRNA-128*	-	[59]
miRNAs with increased expression levels in NMSC	*miR-186–5p*	Targets 3′-UTR P2X7, a ligand-activated membrane channel, which controls cellular proliferation through apoptosis mediation	[60]
	*miR-30e-3p*	Shares cancer-related targets; its reduced expression in elderly advanced OPSC patients correlates with poorer survival, suggesting its key oncogenic roles	[60]
	*miR-875–5p*	-	[60]
	*miR-145–5p*	-	[60]

**Table 3:** Tumor suppressor microRNAs in BCC and SCC carcinogenesis.

	miRNA	Associated Functions	Ref.
miRNAs with decreased expression levels in BCC	*miRNA-34a*	Potential involvement in metastatic progression	[51]
	*miRNA-451a*	Inhibits cell proliferation via cell cycle arrest	[61]
	*miRNA-203*	Potential therapeutic target in BCC management	[62]
	*miRNA-29c*	Inhibits DNA methyltransferases *DNMT3A* and *DNMT3B*	[53,54]
	*miRNA-145*	Targets *EGFR*	[53,54]
	*miRNA-101*	Targets *ING3*	[53,54]
	*miRNA-7b*, *miRNA-141*, *miRNA-9*, *miRNA-200a*, *miRNA-203*, *miRNA-7c*, *miRNA-132*, *miRNA-203*, *miRNA-495*, *miRNA-385*, *miRNA-220a*, *miRNA-30e*, *miRNA-29b*, *miRNA-103*, *miRNA-130a*, *miRNA-144*, *miRNA-381*, *miRNA-452*, *miRNA487b*, *miRNA-494*, *miRNA-590-5p*, *miRNA-139-5p*, *miRNA-140-3p*, *miRNA-572*, *miRNA-638*, *miRNA-2861*, *miRNA-3196*	-	[63]
miRNAs with decreased expression levels in SCC	*miRNA-125b*	Potential therapeutic biomarker: *MMP13* was identified as its direct target.	[64]
	*miRNA-346*	Enhances cSCC proliferation and migration by directly targeting *SRCIN1*	[65]
	*miRNA-361-5p*	Modulates VEGFA expression	[66]
	*miRNA-20a*	May drive CSCC tumorigenesis and progression, serving as a prognostic biomarker for aggressive disease	[67]
	*miRNA-124* *miRNA-214*	Drives *ERK1/2* overexpression, with potential utility for early tumor detection and miRNA-based therapeutics	[68]
	*miRNA-203*	Activates p63 expression, reducing cellular senescence and promoting SCC development	[55]
	*miRNA-26a*	Suppresses *EZH2* expression, a key oncogenic driver	[59]
	*miRNA-145*	Suppresses *FSCN1* in esophageal squamous cell carcinoma	[59]
	*miRNA-378*	Directly targets both *IGF1R* and caspase-3	[59]
	*miRNA-133b*, *miRNA-101*, *miRNA-4324*, *miRNA-136*, *miRNA-204*, *miRNA-497*, *miRNA-29c*, *miRNA-214*	-	[59]
miRNAs with decreased expression levels in NMSC	*miR-30a-5p*	Molecular pathogenesis of cutaneous SCC; found with decreased expression in NMSC	[59]
	*miR-576–3p*		[59]
	*miR-25–3p*		[59]
	*miR-19a-3p*		[59]

**Table 4:** Predisposition biomarkers (genetic susceptibility).

Predisposition Biomarkers	Gene/Proteins Biomarkers		Functions/Role	Ref.
Heritable mutations and associated syndromes	Squamous cell carcinoma (SCC)	XP gene mutations (XPA-G; XPV) and Xeroderma pigmentosum syndrome	Nucleotide excision repair (NER). Replication of damaged DNA on the leading strand	[151]
		BLM (Bloom syndrome, RecQ-like helicase) and Bloom syndrome	DNA-stimulated ATPase and ATP-dependent DNA helicase activities	[151]
		TP53 (Tumor protein p53) and Li–Fraumeni syndrome	Tumor suppressor gene. Cell cycle arrest, apoptosis, senescence, DNA repair, or changes in metabolism	[25]
		*TGFBR1* (Transforming growth factor beta receptor1) and Ferguson–Smith syndrome	Growth factor signaling	[151]
		*COL7A1* (Collagen type VII alpha 1 chain) and recessive dystrophic epidermolysis bullosa (RDEB) syndrome	Anchors fibril between the external epithelia and the underlying stroma	[151]
		*FERMT1* (Fermitin family member 1) and Kindler syndrome	Integrin signaling and linkage of the actin cytoskeleton to the extracellular matrix	[25]
		*MLH1* (MutL homolog 1) and *MSH2* (mutS homolog 2) and Muir–Torre syndrome	DNA repair	[25]
		*DKC1* (Dyskerin pseudouridine synthase 1), *TERC* (Telomerase RNA component), TINF2 (TERF1-interacting nuclear factor 2), *NHP2/NOLA2* (NHP2 ribonucleoprotein/H/ACA RNP complex subunit 2), *NOP10/NOLA3* (NOP10 ribonucleoprotein/H/ACA RNP complex subunit 3), *TERT* (telomerase reverse transcriptase), *WRAP53* (WD repeat containing antisense to TP53) and dyskeratosis congenital syndrome	Telomere maintenance	[25,151]
		TYR (tyrosinase), TYRP1 (tyrosinase-related protein 1), OCA2 (OCA2 melanosomal transmembrane protein), MATP/OCA4 (macrodomain Ter protein/OCA4 melanosomal transmembrane protein and oculocutaneous albinism syndrome	Melanin synthesis	[25,151]
		FANCA, FANC, FANCC, FANCD1, FANCD2, FANCE, FAN, ANCG, FANCI, FANCJ, FANL, FANC, FANCN (Fanconi anemia group protein homolog) and Fanconi anemia syndrome	DNA repair	[151]
		*PTEN* (phosphatase and tensin homolog) and Cowden syndrome	Tumor suppressor gene	[25]
		*RECQL4* (RecQ-like helicase 4) and Rothmund–Thomson syndrome	DNA helicase	[25]
		*WRN* (WRN RecQ-like helicase) and Werner syndrome	DNA helicase	[25]
	Basal cell carcinoma (BCC)	*PTCH* (patched 1) and Gorlin syndrome (basal cell nevus syndrome)	Hedgehog signaling pathway activation. Promotes cell growth and differentiation	[25]
		Skin type	Type1(melanocompromised) and type VI (black skin, melanoprotected)	[152,153]

**Table 5:** Diagnostic, prognostic and predictive biomarkers for NMSC.

Biomarkers	Gene/Proteins Biomarkers		Functions/Role	Ref.
Diagnostic biomarkers	EPCAM (Ber-EP4) (epithelial cell adhesion molecule)		Used to distinguish basal cell carcinoma from squamous cell carcinoma	[154]
Prognostic biomarkers	Tumor microenvironment (TME)	CAFs (cancer-associated fibroblasts)	Promotes tumor growth and metastasis	[155]
		Immune and inflammatory cells: cytokines/growth factors/chemokines/receptors: IFN-γ, TNF-α, IL-10, IL-12, IL-16, IL-17, IL-21, IL-22, IL-2, TGF-β, VEGF-C, CXCR3, CXCL9, CXCL10, CXCL11	Cell proliferation, tumorigenesis and metastasis	[156,157,158,159]
		MMP-7, MMP-9 (matrix metallopeptidase)	Angiogenesis, tumor growth initiation and invasion	[160,161]
	Inflammatory biomarkers	E-cadherin, vimentin, Ki-67 antigen, involucrin, Krt8,18 (keratin 8,18)	Promotes cell–cell adhesion, invasion and metastasis.	[162,163,164]
		CFH (complement factor H)	Proliferation and migration	[165,166]
		FHL-1 (factor-H-like protein-1)	Proliferation and migration	[166]
		Serpin A1	Tumorigenesis	[167]
	Gene alteration (mutations/expression profile/signaling pathways)	TP53 (tumor protein p53) mutation	Apoptosis, cell cycle arrest and senescence	[168]
		CDKN2A (cyclin dependent kinase inhibitor 2A) mutation	Cell cycle progression and senescence	[42]
		RAS mutation	Tumorigenesis and metastasis	[169]
		TGFBR1 (transforming growth factor beta receptor 1) mutation	Cell proliferation survival, invasion, tumor heterogeneity and drug resistance	[170,171]
		NOTCH1/2 mutations	Cell differentiation and morphogenesis	[172,173]
		Loss of heterozygosity (LOH) of adenomatous polyposis coli (APC) gene	Cell migration and adhesion, transcriptional activation (e.g., oncogene activation such as c-Myc and Cyclin D1)	[162]
		PTEN (phosphatase and tensin homolog) expression	Tumor suppressor; negative regulation of AKT/PKB signaling pathway	[174]
		c-myc expression	Cell proliferation and tumorigenesis	[175]
		FOXM1 (forkhead box M1) expression	Tumor cell proliferation	[25,176]
		S100A7 (S100 calcium binding protein A7) expression	Cell proliferation, differentiation, metastasis	[177]
		EphB2 expression	Cell proliferation, tumoral migration and invasion	[168]
		EGFR (epidermal growth factor receptor) expression	Metastasis	[178]
		GLI-1 (glioma-associated oncogene transcription factors) expression	Cell differentiation, proliferation, survival	[179]
		MAPK signaling pathway	Cell proliferation, migration, invasion.	[180,181]
		Wnt/beta-catenin signaling pathway	Critical role in cancer stem cells maintenance in epidermal tumors	[182]
		PI3/AKT-mTOR signaling pathway	Cell proliferation, tumor growth and migration.	[183]
Predictive biomarkers (targeted therapies)	PD-L1 (programmed death-ligand 1)			[157,184]
	PI3K (phosphoinositide 3-kinase)			[185]
	mTOR (mechanistic target of rapamycin kinase)			[186]
	CTLA-4 (cytotoxic T-lymphocyte-associated protein 4)			[187]
	PTCH-1/SMO (patched 1/smoothened, frizzled class receptor)			[188,189]

## Data Availability

No new data were created or analyzed in this study. Data sharing is not applicable to this article.

## References

[1] Nehal K.S., Bichakjian C.K. (2018). Update on keratinocyte carcinomas. N. Engl. J. Med..

[2] Ferlay J., Ervik M., Lam F., Laversanne M., Colombet M., Mery L., Piñeros M., Znaor A., Soerjomataram I., Bray F. (2024). Global Cancer Observatory: Cancer Today.

[3] Zink A. (2017). Non-melanoma skin cancer: Pathogenesis, prevalence and prevention. Der Hautarzt.

[4] Boukamp P. (2005). Non-melanoma skin cancer: What drives tumor development and progression?. Carcinogenesis.

[5] Siegel J., Korgavkar K., Weinstock M. (2017). Current perspective on actinic keratosis: A review. Br. J. Dermatol..

[6] Neagu M., Constantin C., Caruntu C., Dumitru C., Surcel M., Zurac S. (2019). Inflammation: A key process in skin tumorigenesis. Oncol. Lett..

[7] Kwasniak L.A., Garcia-Zuazaga J. (2011). Basal cell carcinoma: Evidence-based medicine and review of treatment modalities. Int. J. Dermatol..

[8] Lucas R., McMichael T., Smith W., Armstrong B. (2006). Solar ultraviolet radiation. Global Burden of Disease from Solar Ultraviolet Radiation.

[9] National Comprehensive Cancer Network, Inc. https://www.nccn.org/patients/guidelines/content/PDF/squamous_cell-patient.pdf.

[10] Huang C.C., Boyce S.M. (2004). Surgical margins of excision for basal cell carcinoma and squamous cell carcinoma. Semin. Cutan. Med. Surg..

[11] Rodriguez-Vigil T., Vázquez-López F., Perez-Oliva N. (2007). Recurrence rates of primary basal cell carcinoma in facial risk areas treated with curettage and electrodesiccation. J. Am. Acad. Dermatol..

[12] Yu S.H., Bordeaux J.S., Baron E.D. (2014). The immune system and skin cancer. Adv. Exp. Med. Biol..

[13] Berg D., Otley C.C. (2002). Skin cancer in organ transplant recipients: Epidemiology, pathogenesis, and management. J. Am. Acad. Dermatol..

[14] Samarasinghe V., Madan V. (2012). Nonmelanoma skin cancer. J. Cutan. Aesthetic Surg..

[15] Samarasinghe V., Madan V., Lear J.T. (2011). Focus on Basal cell carcinoma. J. Ski. Cancer.

[16] Smeets N.W., Kuijpers D.I., Nelemans P., Ostertag J.U., Verhaegh M.E., Krekels G.A., Neumann H.A. (2004). Mohs’ micrographic surgery for treatment of basal cell carcinoma of the face--results of a retrospective study and review of the literature. Br. J. Dermatol..

[17] Zak-Prelich M., Narbutt J., Sysa-Jedrzejowska A. (2004). Environmental risk factors predisposing to the development of basal cell carcinoma. Dermatol. Surg..

[18] Narayanan D.L., Saladi R.N., Fox J.L. (2010). Ultraviolet radiation and skin cancer. Int. J. Dermatol..

[19] Grigore O., Mihailescu A.I., Solomon I., Boda D., Caruntu C. (2019). Role of stress in modulation of skin neurogenic inflammation. Exp. Ther. Med..

[20] D’Orazio J., Jarrett S., Amaro-Ortiz A., Scott T. (2013). UV radiation and the skin. Int. J. Mol. Sci..

[21] Kathpalia V.P., Mussak E.N., Chow S.S., Lam P.H., Skelley N., Time M., Markelewicz R.J., Kanduc D., Lomas L., Xiang Z. (2006). Genome-wide transcriptional profiling in human squamous cell carcinoma of the skin identifies unique tumor-associated signatures. J. Dermatol..

[22] Naldi L., DiLandro A., D’Avanzo B., Parazzini F. (2000). Host-related and environmental risk factors for cutaneous basal cell carcinoma: Evidence from an Italian case-control study. J. Am. Acad. Dermatol..

[23] Fine J.D., Johnson L.B., Weiner M., Li K.P., Suchindran C. (2009). Epidermolysis bullosa and the risk of life-threatening cancers: The National EB Registry experience, 1986–2006. J. Am. Acad. Dermatol..

[24] Binstock M., Hafeez F., Metchnikoff C., Arron S.T. (2014). Single-nucleotide polymorphisms in pigment genes and nonmelanoma skin cancer predisposition: A systematic review. Br. J. Dermatol..

[25] Harwood C.A., Proby C.M., Inman G.J., Leigh I.M. (2016). The Promise of Genomics and the Development of Targeted Therapies for Cutaneous Squamous Cell Carcinoma. Acta Derm. Venereol..

[26] Ruczinski I., Jorgensen T.J., Shugart Y.Y., Schaad Y.B., Kessing B., Hoffman-Bolton J., Helzlsouer K.J., Kao W.H., Wheless L., Francis L. (2012). A population-based study of DNA repair gene variants in relation to non-melanoma skin cancer as a marker of a cancer-prone phenotype. Carcinogenesis.

[27] Powers J.M., Murphy G., Ralph N., O’Gorman S.M., Murphy J.E. (2016). Mitochondrial DNA deletion percentage in sun exposed and non sun exposed skin. J. Photochem. Photobiol. B.

[28] Parr R.L., Dakubo G.D., Thayer R.E., McKenney K., Birch-Machin M.A. (2006). Mi-to-chondrial DNA as a po-ten-tial tool for early can-cer de-tec-tion. Hum. Genom..

[29] Hanahan D. (2022). Hallmarks of Cancer: New Dimensions. Cancer Discov..

[30] Botchkarev V.A., Gdula M.R., Mardaryev A.N., Sharov A.A., Fessing M.Y. (2012). Epigenetic regulation of gene expression in keratinocytes. J. Investig. Dermatol..

[31] Penta D., Somashekar B.S., Meeran S.M. (2018). Epigenetics of skin cancer: Interventions by selected bioactive phytochemicals. Photodermatol. Photoimmunol. Photomed..

[32] Kashyap M.P., Sinha R., Mukhtar M.S., Athar M. (2022). Epigenetic regulation in the pathogenesis of non-melanoma skin cancer. Semin. Cancer Biol..

[33] Rodríguez-Paredes M., Esteller M. (2011). Cancer epigenetics reaches mainstream oncology. Nat. Med..

[34] Pérez R.F., Tejedor J.R., Bayón G.F., Fernández A.F., Fraga M.F. (2018). Distinct chromatin signatures of DNA hypomethylation in aging and cancer. Aging Cell.

[35] Toyota M., Suzuki H. (2010). Epigenetic drivers of genetic alterations. Adv. Genet..

[36] Stamatelli A., Vlachou C., Aroni K., Papassideri I., Patsouris E., Saetta A.A. (2014). Epigenetic alterations in sporadic basal cell carcinomas. Arch. Dermatol. Res..

[37] Brinkhuizen T., van den Hurk K., Winnepenninckx V.J., de Hoon J.P., van Marion A.M., Veeck J., van Engeland M., van Steensel M.A. (2012). Epigenetic changes in Basal Cell Carcinoma affect SHH and WNT signaling components. PLoS ONE.

[38] Takeuchi T., Liang S.B., Ohtsuki Y. (2002). Downregulation of expression of a novel cadherin molecule, T-cadherin, in basal cell carcinoma of the skin. Mol. Carcinog..

[39] Goldberg M., Rummelt C., Laerm A., Helmbold P., Holbach L.M., Ballhausen W.G. (2006). Epigenetic silencing contributes to frequent loss of the fragile histidine triad tumour suppressor in basal cell carcinomas. Br. J. Dermatol..

[40] Lodygin D., Yazdi A.S., Sander C.A., Herzinger T., Hermeking H. (2003). Analysis of 14-3-3sigma expression in hyperproliferative skin diseases reveals selective loss associated with CpG-methylation in basal cell carcinoma. Oncogene.

[41] Greenberg E.S., Chong K.K., Huynh K.T., Tanaka R., Hoon D.S. (2014). Epigenetic biomarkers in skin cancer. Cancer Lett..

[42] Brown V.L., Harwood C.A., Crook T., Cronin J.G., Kelsell D.P., Proby C.M. (2004). p16INK4a and p14ARF tumor suppressor genes are commonly inactivated in cutaneous squamous cell carcinoma. J. Investig. Dermatol..

[43] Murao K., Kubo Y., Ohtani N., Hara E., Arase S. (2006). Epigenetic abnormalities in cutaneous squamous cell carcinomas: Frequent inactivation of the RB1/p16 and p53 pathways. Br. J. Dermatol..

[44] Toll A., Salgado R., Espinet B., Díaz-Lagares A., Hernández-Ruiz E., Andrades E., Sandoval J., Esteller M., Pujol R.M., Hernández-Muñoz I. (2016). MiR-204 silencing in intraepithelial to invasive cutaneous squamous cell carcinoma progression. Mol. Cancer.

[45] O’Brien J., Hayder H., Zayed Y., Peng C. (2018). Overview of MicroRNA Biogenesis, Mechanisms of Actions, and Circulation. Front. Endocrinol..

[46] Tamas T., Baciut M., Nutu A., Bran S., Armencea G., Stoia S., Manea A., Crisan L., Opris H., Onisor F. (2021). Is miRNA Regulation the Key to Controlling Non-Melanoma Skin Cancer Evolution?. Genes.

[47] Green A.C., Olsen C.M. (2017). Cutaneous squamous cell carcinoma: An epidemiological review. Br. J. Dermatol..

[48] Parekh V., Seykora J.T. (2017). Cutaneous Squamous Cell Carcinoma. Clin. Lab. Med..

[49] Heffelfinger C., Ouyang Z., Engberg A., Leffell D.J., Hanlon A.M., Gordon P.B., Zheng W., Zhao H., Snyder M.P., Bale A.E. (2012). Correlation of Global MicroRNA Expression with Basal Cell Carcinoma Subtype. G3 Genes Genomes Genet..

[50] Farzan S.F., Karagas M.R., Christensen B.C., Li Z., Kuriger J.K., Nelson H.H. (2014). New Hampshire Skin Cancer Study. RNASEL and MIR146A SNP-SNP interaction as a susceptibility factor for non-melanoma skin cancer. PLoS ONE.

[51] Chang J., Tran D.C., Zhu G.A., Li R., Whitson R., Kim Y.H., Gupta A., Afshari A., Antes T., Spitale R.C. (2017). Initial in vitro functional characterization of serum exosomal microRNAs from patients with metastatic basal cell carcinoma. Br. J. Dermatol..

[52] Al-Eryani L., Jenkins S.F., States V.A., Pan J., Malone J.C., Rai S.N., Galandiuk S., Giri A.K., States J.C. (2018). miRNA expression profiles of premalignant and malignant arsenic-induced skin lesions. PLoS ONE.

[53] Sand M., Skrygan M., Sand D., Georgas D., Hahn S.A., Gambichler T., Altmeyer P., Bechara F.G. (2012). Expression of microRNAs in basal cell carcinoma. Br. J. Dermatol..

[54] Sand M., Sand D., Altmeyer P., Bechara F.G. (2012). MicroRNA in non-melanoma skin cancer. Cancer Biomark..

[55] Dziunycz P., Iotzova-Weiss G., Eloranta J.J., Lauchli S., Hafner J., French L.E., Altmeyer P., Bechara F.G. (2010). Squamous cell carcinoma of the skin shows a distinct microRNA profile modulated by UV radiation. J. Investig. Dermatol..

[56] Gong Z.H., Zhou F., Shi C., Xiang T., Zhou C.K., Wang Q.Q., Jiang Y.S., Gao S.F. (2019). miRNA-221 promotes cutaneous squamous cell carcinoma progression by targeting PTEN. Cell Mol. Biol. Lett..

[57] Olasz E.B., Seline L.N., Schock A.M., Duncan N.E., Lopez A., Lazar J., Flister M.J., Lu Y., Liu P., Sokumbi O. (2015). MicroRNA-135b Regulates Leucine Zipper Tumor Suppressor 1 in Cutaneous Squamous Cell Carcinoma. PLoS ONE.

[58] Zhou M., Liu W., Ma S., Cao H., Peng X., Guo L., Zhou X., Zheng L., Guo L., Wan M. (2013). A novel onco-miR-365 induces cutaneous squamous cell carcinoma. Carcinogenesis.

[59] Sand M., Skrygan M., Georgas D., Sand D., Hahn S.A., Gambichler T., Altmeyer P., Bechara F.G. (2012). Microarray analysis of microRNA expression in cutaneous squamous cell carcinoma. J. Dermatol. Sci..

[60] Balci S., Ayaz L., Gorur A., Yildirim Yaroglu H., Akbayir S., Dogruer Unal N., Bulut B., Tursen U., Tamer L. (2016). microRNA profiling for early detection of nonmelanoma skin cancer. Clin. Exp. Dermatol..

[61] Sun H., Jiang P. (2018). MicroRNA-451a acts as tumor suppressor in cutaneous basal cell carcinoma. Mol. Genet. Genom. Med..

[62] Sonkoly E., Lov J., Xu N., Meisgen F., Wei T., Brodin P., Jacks V., Kasper M., Shimokawa T., Harada M. (2012). MicroRNA-203 functions as a tumor suppressor in basal cell carcinoma. Oncogenesis.

[63] Wan C., Li Y. (2020). Integrative analysis of mRNA-miRNA-TFs reveals the key regulatory connections involved in basal cell carcinoma. Arch. Dermatol. Res..

[64] Xu N., Zhang L., Meisgen F., Harada M., Heilborn J., Homey B., Grandér D., Ståhle M., Sonkoly E., Pivarcsi A. (2012). MicroRNA-125b down-regulates matrix metallopeptidase 13 and inhibits cutaneous squamous cell carcinoma cell proliferation, migration, and invasion. J. Biol. Chem..

[65] Chen B., Pan W., Lin X., Hu Z., Jin Y., Chen H., Ma G., Qiu Y., Chang L., Hua C. (2016). MicroRNA-346 functions as an oncogene in cutaneous squamous cell carcinoma. Tumor Biol..

[66] Kanitz A., Imig J., Dziunycz P.J., Primorac A., Galgano A., Hofbauer G.F.L., Gerber A.P., Detmar M. (2012). The expression levels of microRNA-361-5p and its target VEGFA are inversely correlated in human cutaneous squamous cell carcinoma. PLoS ONE.

[67] Zhang L., Xiang P., Han X., Wu L., Li X., Xiong Z. (2015). Decreased expression of microRNA-20a promotes tumor progression and predicts poor prognosis of cutaneous squamous cell carcinoma. Int. J. Clin. Exp. Pathol..

[68] Yamane K., Jinnin M., Etoh T., Kobayashi Y., Shimozono N., Fukushima S., Masuguchi S., Maruo K., Inoue Y., Ishihara T. (2013). Down-regulation of miR-124/-214 in cutaneous squamous cell carcinoma mediates abnormal cell proliferation via the induction of ERK. J. Mol. Med..

[69] Mattick J.S., Amaral P.P., Carninci P., Carpenter S., Chang H.Y., Chen L.L., Chen R., Dean C., Dinger M.E., Fitzgerald K.A. (2023). Long non-coding RNAs: Definitions, functions, challenges and recommendations. Nat. Rev. Mol. Cell Biol..

[70] Piipponen M., Heino J., Kähäri V.M., Nissinen L. (2018). Long non-coding RNA PICSAR decreases adhesion and promotes migration of squamous carcinoma cells by downregulating α2β1 and α5β1 integrin expression. Biol. Open.

[71] Mei X.L., Zhong S. (2019). Long noncoding RNA LINC00520 prevents the progression of cutaneous squamous cell carcinoma through the inactivation of the PI3K/Akt signaling pathway by downregulating EGFR. Chin. Med. J..

[72] Mancini M., Cappello A., Pecorari R., Lena A.M., Montanaro M., Fania L., Ricci F., Di Lella G., Piro M.C., Abeni D. (2021). Involvement of transcribed lncRNA uc.291 and SWI/SNF complex in cutaneous squamous cell carcinoma. Discov. Oncol..

[73] Dehcheshmeh I.S., Karimi M.M., Jafarisani M. (2018). Increased Expression of CCAT2 LncRNA in Non-Melanoma Skin Cancer. Shahroud J. Med. Sci..

[74] El Derbaly S.A., Hamouda M.A.F., Abdallah Elnahas M., Mahfouz Hamed1 A., Abd El Gayed E.M. (2025). Unveiling the Role of lncRNA BBOX1-AS1, HOXB7, and IGF2BP1 in Non-Melanocytic Skin Tumors: Potential Biomarkers for Diagnosis. Egypt. J. Hosp. Med..

[75] Ma Y., Madupu R., Karaoz U., Nossa C.W., Yang L., Yooseph S., Yachimski P.S., Brodie E.L., Nelson K.E., Pei Z. (2014). Human papillomavirus community in healthy persons, defined by metagenomics analysis of human microbiome project shotgun sequencing data sets. J. Virol..

[76] Bzhalava D., Mühr L.S., Lagheden C., Ekström J., Forslund O., Dillner J., Hultin E. (2014). Deep sequencing extends the diversity of human papillomaviruses in human skin. Sci. Rep..

[77] Antonsson A., Karanfilovska S., Lindqvist P.G., Hansson B.G. (2003). General acquisition of human papillomavirus infections of skin occurs in early infancy. J. Clin. Microbiol..

[78] Bruggink S.C., de Koning M.N., Gussekloo J., Egberts P.F., Ter Schegget J., Feltkamp M.C., Bavinck J.N., Quint W.G., Assendelft W.J., Eekhof J.A. (2012). Cutaneous wart-associated HPV types: Prevalence and relation with patient characteristics. J. Clin. Virol. Off. Publ. Pan Am. Soc. Clin. Virol..

[79] Rahman S., Pierce Campbell C.M., Rollison D.E., Wang W., Waterboer T., Michel A., Pawlita M., Villa L.L., Lazcano Ponce E., Borenstein A.R. (2016). Seroprevalence and Associated Factors of 9-Valent Human Papillomavirus (HPV) Types among Men in the Multinational HIM Study. PLoS ONE.

[80] Hampras S.S., Giuliano A.R., Lin H.Y., Fisher K.J., Abrahamsen M.E., Sirak B.A., Iannacone M.R., Gheit T., Tommasino M., Rollison D.E. (2014). Natural history of cutaneous human papillomavirus (HPV) infection in men: The HIM study. PLoS ONE.

[81] Hasche D., Vinzón S.E., Rösl F. (2018). Cutaneous Papillomaviruses and Non-melanoma Skin Cancer: Causal Agents or Innocent Bystanders?. Front. Microbiol..

[82] Ally M.S., Tang J.Y., Arron S.T. (2013). Cutaneous human papillomavirus infection and Basal cell carcinoma of the skin. J. Investig. Dermatol..

[83] And Ersson K., Michael K.M., Luostarinen T., Waterboer T., Gislefoss R., Hakulinen T., Forslund O., Pawlita M., Dillner J. (2012). Prospective study of human papillomavirus seropositivity and risk of nonmelanoma skin cancer. Am. J. Epidemiol..

[84] And Ersson K., Waterboer T., Kirnbauer R., Slupetzky K., Iftner T., de Villiers E.M., Forslund O., Pawlita M., Dillner J. (2008). Seroreactivity to cutaneous human papillomaviruses among patients with nonmelanoma skin cancer or benign skin lesions. Cancer Epidemiol. Biomark. Prev..

[85] Sandoval-Clavijo A., Martí-Martí I., Ferrándiz-Pulido C., Verdaguer-Faja J., Jaka A., Toll A. (2025). Human Papillomavirus-Related Cutaneous Squamous Cell Carcinoma. Cancers.

[86] Iannacone M.R., Gheit T., Waterboer T., Giuliano A.R., Messina J.L., Fenske N.A., Cherpelis B.S., Sondak V.K., Roetzheim R.G., Ferrer-Gil S. (2013). Case–Control Study of Cutaneous Human Papillomavirus Infection in Basal Cell Carcinoma of the Skin. J. Investig. Dermatol..

[87] Hasche D., Stephan S., Braspenning-Wesch I., Mikulec J., Niebler M., Gröne H., Flechtenmacher C., Akgül B., Rösl F., Vinzón S.E. (2017). The interplay of UV and cutaneous papillomavirus infection in skin cancer development. PLoS Pathog..

[88] IARC Working Group on the Evaluation of Carcinogenic Risks to Humans (1992). Solar and Ultraviolet Radiation.

[89] Young A.R., Giacomoni P.U., Jori G., Hader D.P. (2007). Chapter 1: Damage from acute vs chronic solar exposure. Biophysical and Physiological Effects of Solar Radiation on Human Skin.

[90] Hunter N., Haylock R. (2026). The risk of basal and squamous cell carcinomas of the skin cancer incidence and external radiation in the updated National Registry for radiation workers cohort in the UK. Int. J. Cancer..

[91] World Health Organization (2021). The Effect of Occupational Exposure to Solar Ultraviolet Radiation on Malignant Skin Melanoma and Nonmelanoma Skin Cancer: A Systematic Review and Meta-Analysis from the WHO/ILO Joint Estimates of the Work-Related Burden of Disease and Injury.

[92] Saitoh Y., Miyanishi A., Mizuno H., Kato S., Aoshima H., Kokubo K., Miwa N. (2011). Su-per-highly hydroxylated fullerene derivative protects human keratinocytes from UV-induced cell injuries together with the decreases in intracellular ROS generation and DNA damages. J. Photochem. Photobiol..

[93] Kim Y., He Y.Y. (2014). Ultraviolet radiation-induced non-melanoma skin cancer: Regulation of DNA damage repair and inflammation. Genes Dis..

[94] Calzavara-Pinton P., Ortel B., Venturini M. (2015). Non-melanoma skin cancer, sun exposure and sun protection. G. Ital. Dermatol. Venereol..

[95] López-Camarillo C., Ocampo E.A., Casamichana M.L., Pérez-Plasencia C., Alvarez-Sánchez E., Marchat L.A. (2012). Protein kinases and transcription factors activation in response to UV-radiation of skin: Implications for carcinogenesis. Int. J. Mol. Sci..

[96] Von Schuckmann L.A., Law M.H., Montgomery G.W., Green A.C., Van Der Pols J.C. (2016). Vitamin D Pathway Gene Polymorphisms and Keratinocyte Cancers: A Nested Case-Control Study and Meta-Analysis. Anticancer Res..

[97] IARC Working Group on the Evaluation of Carcinogenic Risks to Humans (1991). Occupational exposures in insecticide application, and some pesticides. IARC Monographs on the Evaluation of Carcinogenic Risks to Humans.

[98] International Agency for Research on Cancer (IARC) List of Classifications by Cancer Sites with Sufficient or Limited Evidence in Humans. IARC Monographs.

[99] Parker E.R. (2020). The influence of climate change on skin cancer incidence—A review of the evidence. Int. J. Womens Dermatol..

[100] de Martel C., Ferlay J., Franceschi S., Vignat J., Bray F., Forman D., Plummer M. (2012). Global burden of cancers attributable to infections in 2008: A review and synthetic analysis. Lancet Oncol..

[101] Rahman M.M., Islam M.R., Shohag S., Ahasan M.T., Sarkar N., Khan H., Hasan A.M., Cavalu S., Rauf A. (2022). Microbiome in cancer: Role in carcinogenesis and impact in therapeutic strategies. Biomed. Pharmacother..

[102] Nakatsuji T., Chen T.H., Butcher A.M., Trzoss L.L., Nam S.-J., Shirakawa K.T., Zhou W., Oh J., Otto M., Fenical W. (2018). A commensal strain of Staphylococcus epidermidis protects against skin neoplasia. Sci. Adv..

[103] Godar D.E. (2021). UV and Reactive Oxygen Species Activate Human Papillomaviruses Causing Skin Cancers. Curr. Probl. Dermatol..

[104] Egert M., Simmering R., Riedel C.U. (2017). The Association of the Skin Microbiota with Health, Immunity, and Disease. Clin. Pharmacol. Ther..

[105] Squarzanti D.F., Zavattaro E., Pizzimenti S., Amoruso A., Savoia P., Azzimonti B. (2020). Non-Melanoma Skin Cancer: News from microbiota research. Crit. Rev. Microbiol..

[106] Kullander J., Forslund O., Dillner J. (2009). Staphylococcus aureus and squamous cell carcinoma of the skin. Cancer Epidemiol. Biomark. Prev..

[107] Wood D.L.A., Lachner N., Tan J.-M., Tang S., Angel N., Laino A., Linedale R., Cao K.-A.L., Morrison M., Frazer I.H. (2018). A natural history of actinic keratosis and cutaneous squamous cell carcinoma microbiomes. mBio.

[108] Madhusudhan N., Pausan M.R., Halwachs B., Durdević M., Windisch M., Kehrmann J., Patra V., Wolf P., Boukamp P., Moissl-Eichinger C. (2020). Molecular Profiling of Keratinocyte Skin Tumors Links Staphylococcus aureus Overabundance and Increased Human β-Defensin-2 Expression to Growth Promotion of Squamous Cell Carcinoma. Cancers.

[109] Voigt A.Y., Emiola A., Johnson J.S., Fleming E.S., Nguyen H., Zhou W., Tsai K.Y., Fink C., Oh J. (2022). Skin Microbiome Variation with Cancer Progression in Human Cutaneous Squamous Cell Carcinoma. J. Investig. Dermatol..

[110] Byrd A., Belkaid Y., Segre J. (2018). The human skin microbiome. Nat. Rev. Microbiol..

[111] Tomás-Pejó E., González-Fernández C., Greses S., Kennes C., Otero-Logilde N., Veiga M.C., Bolzonella D., Müller B., Passoth V. (2023). Production of Short-Chain Fatty Acids (SCFAs) as Chemicals or Substrates for Microbes to Obtain Biochemicals. Biotechnol. Biofuels Bioprod..

[112] Thomas S.P., Denu J.M. (2021). Short-Chain Fatty Acids Activate Acetyltransferase P300. eLife.

[113] Szabó K., Bolla B.S., Erdei L., Balogh F., Kemény L. (2023). Are the Cutaneous Microbiota a Guardian of the Skin’s Physical Barrier? The Intricate Relationship between Skin Microbes and Barrier Integrity. Int. J. Mol. Sci..

[114] Bolla B.S., Erdei L., Urbán E., Burián K., Kemény L., Szabó K. (2020). Cutibacterium Acnes Reg-ulates the Epidermal Barrier Properties of HPV-KER Human Immortalized Keratinocyte Cultures. Sci. Rep..

[115] Zeng R., Xu H., Liu Y., Du L., Duan Z., Tong J., He Y., Chen Q., Chen X., Li M. (2019). MiR-146a Inhibits Biofilm-Derived Cutibacterium Acnes–Induced Inflammatory Reactions in Human Keratinocytes. J. Investig. Dermatol..

[116] Kreher M.A., Noland M.M.B., Konda S., Longo M.I., Valdes-Rodriguez R. (2023). Risk of melanoma and nonmelanoma skin cancer with immunosuppressants, part I: Calcineurin inhibitors, thiopurines, IMDH inhibitors, mTOR inhibitors, and corticosteroids. J. Am. Acad. Dermatol..

[117] Nardone B., West D.P. (2025). Non-melanoma skin cancer and other adverse events resulting from antihypertensive drug use: What do we know?. Expert Opin. Drug Saf..

[118] Yang K., Marley A., Tang H., Song Y., Tang J.Y., Han J. (2017). Statin use and non-melanoma skin cancer risk: A meta-analysis of rand omized controlled trials and observational studies. Oncotarget.

[119] O’Neill B., Moe S., Korownyk C. (2020). Hydrochlorothiazide and squamous cell carcinoma. Can. Fam. Physician.

[120] Hohl M., Götzinger F., Jäger S., Wagmann L., Tokcan M., Tschernig T., Reichrath J., Federspiel J.M., Boor P., Meyer M.R. (2025). Assessing phototoxic drug properties of hydrochlo-rothiazide using human skin biopsies. Commun. Biol..

[121] Bivona J.J., Patel S., Vajdy M. (2017). Induction of cellular and molecular Immunomodulatory pathways by vitamin E and vitamin C. Expert Opin. Biol. Ther..

[122] Weinstock M.A., Bingham S.F., DiGiovanna J.J., Rizzo A.E., Marcolivio K., Hall R., Eilers D., Naylor M., Kirsner R., Kalivas J. (2012). Tretinoin and the prevention of keratinocyte carcinoma (Basal and squamous cell carcinoma of the skin): A veterans affairs rand omized chemoprevention trial. J. Investig. Dermatol..

[123] Fania L., Mazzanti C., Campione E., Candi E., Abeni D., Dellambra E. (2019). Role of Nicotinamide in Genomic Stability and Skin Cancer Chemoprevention. Int. J. Mol. Sci..

[124] Nazarali S., Kuzel P. (2017). Vitamin B Derivative (Nicotinamide)Appears to Reduce Skin Cancer Risk. Ski. Ther. Lett..

[125] Surjana D., Halliday G.M., Damian D.L. (2010). Role of nicotinamide in DNA damage, mutagenesis, and DNA repair. J. Nucleic Acids.

[126] Chen A.C., Martin A.J., Choy B., Fernández-Peñas P., Dalziell R.A., McKenzie C.A., Scolyer R.A., Dhillon H.M., Vardy J.L., Kricker A. (2015). A Phase 3 Rand omized Trial of Nicotinamide for Skin-Cancer Chemoprevention. N. Engl. J. Med..

[127] Park S.M., Li T., Wu S., Li W.Q., Weinstock M., Qureshi A.A., Cho E. (2017). Niacin intake and risk of skin cancer in US women and men. Int. J. Cancer.

[128] Singh S., Aggarwal B.B. (1995). Activation of transcription factor NF-kappa B is suppressed by curcumin (diferuloylmethane) [corrected]. J. Biol. Chem..

[129] Amiri K.I., Richmond A. (2005). Role of nuclear factor-kappa B in melanoma. Cancer Metastasis Rev..

[130] Kumar A., Dhawan S., Hardegen N.J., Aggarwal B.B. (1998). Curcumin (Diferuloylmethane) inhibition of tumor necrosis factor (TNF)-mediated adhesion of monocytes to endothelial cells by suppression of cell surface expression of adhesion molecules and of nuclear factor-kappaB activation. Biochem. Pharmacol..

[131] Kuttan R., Sudheeran P.C., Josph C.D. (1987). Turmeric and curcumin as topical agents in cancer therapy. Tumori J..

[132] Rhode J., Fogoros S., Zick S., Wahl H., Griffith K.A., Huang J., Liu J.R. (2007). Ginger inhibits cell growth and modulates angiogenic factors in ovarian cancer cells. BMC Complement. Altern. Med..

[133] Nigam N., Bhui K., Prasad S., George J., Shukla Y. (2009). [6]-Gingerol induces reactive oxygen species regulated mitochondrial cell death pathway in human epidermoid carcinoma A431 cells. Chem. Biol. Interact..

[134] Banerjee S., Panda C.K., Das S. (2006). Clove (*Syzygium aromaticum* L.), a potential chemopreventive agent for lung cancer. Carcinogenesis.

[135] Murakami Y., Shoji M., Hanazawa S., Tanaka S., Fujisawa S. (2003). Preventive effect of bis-eugenol, a eugenol ortho dimer, on lipopolysaccharide-stimulated nuclear factor kappa B activation and inflammatory cytokine expression in macrophages. Biochem. Pharmacol..

[136] Nichols J.A., Katiyar S.K. (2010). Skin photoprotection by natural polyphenols: Anti-inflammatory, antioxidant and DNA repair mechanisms. Arch. Dermatol. Res..

[137] Katta R., Brown D.N. (2015). Diet and Skin Cancer: The Potential Role of Dietary Antioxidants in Nonmelanoma Skin Cancer Prevention. J. Ski. Cancer.

[138] Sancheti G., Goyal P.K. (2006). Effect of Rosmarinus officinalis in modulating 7,12-dimethylbenz(a)anthracene induced skin tumorigenesis in mice. Phytother. Res..

[139] Das I., Chakrabarty R.N., Das S. (2004). Saffron can prevent chemically induced skin carcinogenesis in Swiss albino mice. Asian Pac. J. Cancer Prev..

[140] Lee G.R., Jang S.H., Kim C.J., Kim A.R., Yoon D.J., Park N.H., Han I.S. (2014). Capsaicin suppresses the migration of cholangiocarcinoma cells by down-regulating matrix metalloproteinase-9 expression via the AMPK-NF-κB signaling pathway. Clin. Exp. Metastasis.

[141] Sreedhar A., Li J., Zhao Y. (2018). Next-Gen Therapeutics for Skin Cancer: Nutraceuticals. Nutr. Cancer.

[142] Zhou X., Münch G., Wohlmuth H., Afzal S., Kao M.-H. (2022). Synergistic Inhibition of Pro-Inflammatory Pathways by Ginger and Turmeric Extracts in RAW 264.7 Cells. Front. Pharmacol..

[143] Ouyang B., Wu Q., Liang J., Cao H., Xiao J. (2025). Polyphenols-mediated immune regulation: Metabolite-driven epigenetic regulatory mechanisms. Phytomedicine.

[144] Chou P.J., Peter R.M., Shannar A., Pan Y., Dave P.D., Xu J., Sarwar M.S., Kong A.N. (2024). Epigenetics of Dietary Phytochemicals in Cancer Prevention: Fact or Fiction. Cancer J..

[145] Bacaloni S., Agrawal D.K. (2025). Nutrition, Gut Microbiota, and Epigenetics in the Modulation of Immune Response and Metabolic Health. Cardiol. Cardiovasc. Med..

[146] Castelo J., Araujo-Aris S., Barriales D., Tanner Pasco S., Seoane I., Peña-Cearra A., Palacios A., Simó C., Garcia-Cañas V., Khamwong M. (2024). The microbiota metabolite, phloroglucinol, confers long-term protection against in-flammation. Gut Microbes.

[147] Guo X., Li J., Xu J., Zhang L., Huang C., Nie Y., Zhou Y. (2025). Gut microbiota and epigenetic inher-itance: Implications for the development of IBD. Gut Microbes.

[148] Hunt K.M., Srivastava R.K., Elmets C.A., Athar M. (2014). The mechanistic basis of arsenicosis: Pathogenesis of skin cancer. Cancer Lett..

[149] Shen J., Abel E.L., Riggs P.K., Repass J., Hensley S.C., Schroeder L.J., Temple A., Chau A., McClellan S.A., Rho O. (2012). Proteomic and pathway analyses reveal a network of inflammatory genes associated with differences in skin tumor promotion susceptibility in DBA/2 and C57BL/6 mice. Carcinogenesis.

[150] Dusingize J.C., Olsen C.M., Pandeya N.P., Subramaniam P., Thompson B.S., Neale R.E., Green A.C., Whiteman D.C. (2017). Cigarette Smoking and the Risks of Basal Cell Carcinoma and Squamous Cell Carcinoma. J. Investig. Dermatol..

[151] Nikolaou V., Stratigos A.J., Tsao H. (2012). Hereditary nonmelanoma skin cancer. Semin. Cutan. Med. Surg..

[152] Greinert R. (2009). Skin cancer: New markers for better prevention. Pathobiology.

[153] Gandini S., Sera F., Cattaruzza M.S., Pasquini P., Abeni D., Boyle P., Melchi C.F. (2005). Meta-analysis of risk factors for cutaneous melanoma: I. Common and atypical naevi. Eur. J. Cancer.

[154] Beer T.W., Shepherd P., Theaker J.M. (2000). Ber EP4 and epithelial membrane antigen aid distinction of basal cell, squamous cell and basosquamous carcinomas of the skin. Histopathology.

[155] Omland S.H., Wettergren E.E., Mollerup S., Asplund M., Mourier T., Hansen A.J., Gniadecki R. (2017). Cancer associated fibroblasts (CAFs) are activated in cutaneous basal cell carcinoma and in the peritumoural skin. BMC Cancer.

[156] Bridge J.A., Lee J.C., Daud A., Wells J.W., Bluestone J.A. (2018). Cytokines, Chemokines, and Other Biomarkers of Response for Checkpoint Inhibitor Therapy in Skin Cancer. Front. Med..

[157] Nicholas C., Lesinski G.B. (2011). Immunomodulatory cytokines as therapeutic agents for melanoma. Immunotherapy.

[158] Chow M.T., Luster A.D. (2014). Chemokines in cancer. Cancer Immunol. Res..

[159] Byrne S.N., Halliday G.M., Wondrak G.T. (2016). UV-Induced Chemokines as Emerging Targets for Skin Cancer Photochemoprevention. Skin Stress Response Pathways: Environmental Factors and Molecular Opportunities.

[160] Pittayapruek P., Meephansan J., Prapapan O., Komine M., Ohtsuki M. (2016). Role of Matrix Metalloproteinases in Photoaging and Photocarcinogenesis. Int. J. Mol. Sci..

[161] Didona D., Paolino G., Bottoni U., Cantisani C. (2018). Non Melanoma Skin Cancer Pathogenesis Overview. Biomedicines.

[162] Griffin J.R., Wriston C.C., Peters M.S., Lehman J.S. (2013). Decreased expression of intercellular adhesion molecules in acantholytic squamous cell carcinoma compared with invasive well-differentiated squamous cell carcinoma of the skin. Am. J. Clin. Pathol..

[163] Lan Y.J., Chen H., Chen J.Q., Lei Q.H., Zheng M., Shao Z.R. (2014). Immunolocalization of vimentin, keratin 17, Ki-67, involucrin, β-catenin and E-cadherin in cutaneous squamous cell carcinoma. Pathol. Oncol. Res..

[164] Yamashiro Y., Takei K., Umikawa M., Asato T., Oshiro M., Uechi Y., Ishikawa T., Taira K., Uezato H., Kariya K. (2010). Ectopic coexpression of keratin 8 and 18 promotes invasion of transformed keratinocytes and is induced in patients with cutaneous squamous cell carcinoma. Biochem. Biophys. Res. Commun..

[165] Voiculescu V., Calenic B., Ghita M., Lupu M., Caruntu A., Moraru L., Voiculescu S., Ion A., Greabu M., Ishkitiev N. (2016). From Normal Skin to Squamous Cell Carcinoma: A Quest for Novel Biomarkers. Dis. Markers.

[166] Riihilä P.M., Nissinen L.M., Ala-Aho R., Kallajoki M., Grénman R., Meri S., Peltonen S., Peltonen J., Kähäri V.M. (2014). Complement factor H: A biomarker for progression of cutaneous squamous cell carcinoma. J. Investig. Dermatol..

[167] Farshchian M., Kivisaari A., Ala-Aho R., Riihilä P., Kallajoki M., Grénman R., Peltonen J., Pihlajaniemi T., Heljasvaara R., Kähäri V.M. (2011). Serpin peptidase inhibitor clade A member 1 (SerpinA1) is a novel biomarker for progression of cutaneous squamous cell carcinoma. Am. J. Pathol..

[168] Giglia-Mari G., Sarasin A. (2003). TP53 mutations in human skin cancers. Hum. Mutat..

[169] Su F., Viros A., Milagre C., Trunzer K., Bollag G., Spleiss O., Reis-Filho J.S., Kong X., Koya R.C., Flaherty K.T. (2012). RAS mutations in cutaneous squamous-cell carcinomas in patients treated with BRAF inhibitors. N. Engl. J. Med..

[170] Karagas M.R., Waterboer T., Li Z., Nelson H.H., Michael K.M., Bavinck J.N.B., Perry A.E., Spencer S.K., Daling J., Green A.C. (2010). Genus beta human papillomaviruses and incidence of basal cell and squamous cell carcinomas of skin: Population based case-control study. BMJ.

[171] Oshimori N., Oristian D., Fuchs E. (2015). TGF-β promotes heterogeneity and drug resistance in squamous cell carcinoma. Cell.

[172] South A.P., Purdie K.J., Watt S.A., Haldenby S., den Breems N., Dimon M., Arron S.T., Kluk M.J., Aster J.C., McHugh A. (2014). NOTCH1 mutations occur early during cutaneous squamous cell carcinogenesis. J. Investig. Dermatol..

[173] Wang N.J., Sanborn Z., Arnett K.L., Bayston L.J., Liao W., Proby C.M., Leigh I.M., Collisson E.A., Gordon P.B., Jakkula L. (2011). Loss-of-function mutations in Notch receptors in cutaneous and lung squamous cell carcinoma. Proc. Natl. Acad. Sci. USA.

[174] Ming M., Feng L., Shea C.R., Soltani K., Zhao B., Han W., Smart R.C., Trempus C.S., He Y.Y. (2011). PTEN positively regulates UVB-induced DNA damage repair. Cancer Res..

[175] Honeycutt K.A., Waikel R.L., Koster M.I., Wang X.J., Roop D.R. (2010). The effect of c-myc on stem cell fate influences skin tumor phenotype. Mol. Carcinog..

[176] Teh M.T., Hutchison I.L., Costea D.E., Neppelberg E., Liavaag P.G., Purdie K., Harwood C., Wan H., Odell E.W., Hackshaw A. (2013). Exploiting FOXM1-orchestrated molecular network for early squamous cell carcinoma diagnosis and prognosis. Int. J. Cancer.

[177] Qi Z., Li T., Kong F., Li Y., Wang R., Wang J., Xiao Q., Zhang W., Sun S., He D. (2015). The Characteristics and Function of S100A7 Induction in Squamous Cell Carcinoma: Heterogeneity, Promotion of Cell Proliferation and Suppression of Differentiation. PLoS ONE.

[178] Fogarty G.B., Conus N.M., Chu J., McArthur G. (2007). Characterization of the expression and activation of the epidermal growth factor receptor in squamous cell carcinoma of the skin. Br. J. Dermatol..

[179] Varjosalo M., Taipale J. (2008). Hedgehog: Functions and mechanisms. Genes Dev..

[180] Pourreyron C., Chen M., McGrath J.A., Salas-Alanis J.C., South A.P., Leigh I.M. (2014). High levels of type VII collagen expression in recessive dystrophic epidermolysis bullosa cutaneous squamous cell carcinoma keratinocytes increases PI3K and MAPK signalling, cell migration and invasion. Br. J. Dermatol..

[181] Lambert S.R., Mladkova N., Gulati A., Hamoudi R., Purdie K., Cerio R., Leigh I., Proby C., Harwood C.A. (2014). Key differences identified between actinic keratosis and cutaneous squamous cell carcinoma by transcriptome profiling. Br. J. Cancer.

[182] Malanchi I., Peinado H., Kassen D., Hussenet T., Metzger D., Chambon P., Huber M., Hohl D., Cano A., Birchmeier W. (2008). Cutaneous cancer stem cell maintenance is dependent on beta-catenin signalling. Nature.

[183] Cantley L.C. (2002). The phosphoinositide 3-kinase pathway. Science.

[184] Loo K., Tsai K.K., Mahuron K., Liu J., Pauli M.L., Sandoval P.M., Nosrati A., Lee J., Chen L., Hwang J. (2017). Partially exhausted tumor-infiltrating lymphocytes predict response to combination immunotherapy. JCI Insight.

[185] Janus J.M., O’Shaughnessy R.F.L., Harwood C.A., Maffucci T. (2017). Phosphoinositide 3-Kinase-Dependent Signalling Pathways in Cutaneous Squamous Cell Carcinomas. Cancers.

[186] Salgo R., Gossmann J., Schöfer H., Kachel H.G., Kuck J., Geiger H., Kaufmann R., Scheuermann E.H. (2010). Switch to a sirolimus-based immunosuppression in long-term renal transplant recipients: Reduced rate of (pre-)malignancies and nonmelanoma skin cancer in a prospective, rand omized, assessor-blinded, controlled clinical trial. Am. J. Transplant..

[187] Ghafouri-Fard S., Ghafouri-Fard S. (2012). Immunotherapy in nonmelanoma skin cancer. Immunotherapy.

[188] Sekulic A., Migden M.R., Oro A.E., Dirix L., Lewis K.D., Hainsworth J.D., Solomon J.A., Yoo S., Arron S.T., Friedlander P.A. (2012). Efficacy and safety of vismodegib in advanced basal-cell carcinoma. N. Engl. J. Med..

[189] Kish T., Corry L. (2016). Sonidegib (Odomzo) for the Systemic Treatment of Adults with Recurrent, Locally Advanced Basal Cell Skin Cancer. Pharm. Ther..

[190] Abramson A.K., Krasny M.J., Goldman G.D. (2013). Tangential shave removal of basal cell carcinoma. Dermatol. Surg..

[191] Lv R., Sun Q. (2017). A Network Meta-Analysis of Non-Melanoma Skin Cancer (NMSC) Treatments: Efficacy and Safety Assessment. J. Cell. Biochem..

[192] Cheraghi N., Cognetta A., Goldberg D. (2017). Radiation Therapy in Dermatology: Non-Melanoma Skin Cancer. J. Drugs Dermatol..

[193] Micali G., Lacarrubba F., Nasca M.R., Ferraro S., Schwartz R.A. (2014). Topical pharmacotherapy for skin cancer: Part II. Clinical applications. J. Am. Acad. Dermatol..

[194] Russo I., Sernicola A., Alaibac M. (2019). Recent advances in localized immunotherapy of skin cancers. Immunotherapy.

[195] Raasch B. (2009). Management of superficial basal cell carcinoma: Focus on imiquimod. Clin. Cosmet. Investig. Dermatol..

[196] Huang S.M., Harari P.M. (1999). Epidermal growth factor receptor inhibition in cancer therapy: Biology, rationale and preliminary clinical results. Investig. New Drugs.

[197] Maubec E., Petrow P., Scheer-Senyarich I., Duvillard P., Lacroix L., Gelly J., Certain A., Duval X., Crickx B., Buffard V. (2011). Phase II study of cetuximab as first-line single-drug therapy in patients with unresectable squamous cell carcinoma of the skin. J. Clin. Oncol..

[198] Saran A. (2010). Basal cell carcinoma and the carcinogenic role of aberrant Hedgehog signaling. Future Oncol..

[199] Katoh Y., Katoh M. (2009). Hedgehog target genes: Mechanisms of carcinogenesis induced by aberrant hedgehog signaling activation. Curr. Mol. Med..

[200] Schneider S., Thurnher D., Kloimstein P., Leitner V., Petzelbauer P., Pammer J., Brunner M., Erovic B.M. (2011). Expression of the Sonic hedgehog pathway in squamous cell carcinoma of the skin and the mucosa of the head and neck. Head Neck.

[201] Geng L., Cuneo K.C., Cooper M.K., Wang H., Sekhar K., Fu A., Hallahan D.E. (2007). Hedgehog signaling in the murine melanoma microenvironment. Angiogenesis.

[202] Marzuka A.G., Book S.E. (2015). Basal cell carcinoma: Pathogenesis, epidemiology, clinical features, diagnosis, histopathology, and management. Yale J. Biol. Med..

[203] Otsuka A., Levesque M.P., Dummer R., Kabashima K. (2015). Hedgehog signaling in basal cell carcinoma. J. Dermatol. Sci..

[204] Gu H., Li X.U., Zhou C., Wen Y., Shen Y., Zhou L., Li J. (2015). Effects and mechanisms of blocking the hedgehog signaling pathway in human gastric cancer cells. Oncol. Lett..

[205] Robarge K.D., Brunton S.A., Castanedo G.M., Cui Y., Dina M.S., Goldsmith R., Gould S.E., Guichert O., Gunzner J.L., Halladay J. (2009). GDC-0449-a potent inhibitor of the hedgehog pathway. Bioorg. Med. Chem. Lett..

[206] FDA-ERIVEDGE^®^ Prescribing Information. https://www.accessdata.fda.gov/drugsatfda_docs/label/2012/203388lbl.pdf.

[207] (2019). EMA—European Public Assessment Report (EPAR) for Erivedge^®^. https://www.ema.europa.eu/en/documents/assessment-report/erivedge-epar-public-assessment-report_en.pdf.

[208] (2019). EMA—European Public Assessment Report (EPAR) for Odomzo^®^. https://www.ema.europa.eu/en/documents/assessment-report/odomzo-epar-public-assessment-report_en.pdf.

[209] National Comprehensive Cancer Network https://www.nccn.org/professionals/physician_gls/pdf/nmsc_blocks.pdf.

[210] Brinkhuizen T., Reinders M.G., van Geel M., Hendriksen A.J., Paulussen A.D., Winnepenninckx V.J., Keymeulen K.B., Soetekouw P.M., van Steensel M.A., Mosterd K. (2014). Acquired resistance to the Hedgehog pathway inhibitor vismodegib due to smoothened mutations in treatment of locally advanced basal cell carcinoma. J. Am. Acad. Dermatol..

[211] Danial C., Sarin K.Y., Oro A.E., Chang A.L. (2016). An Investigator-Initiated Open-Label Trial of Sonidegib in Advanced Basal Cell Carcinoma Patients Resistant to Vismodegib. Clin. Cancer Res..

[212] Ascierto P.A., Schadendorf D. (2019). Immunotherapy in non-melanoma skin cancer: Updates and new perspectives. Drugs Context.

[213] Berman B., Perez O.A., Zell D. (2006). Immunological strategies to fight skin cancer. Ski. Ther. Lett..

[214] Tarhini A.A., Gogas H., Kirkwood J.M. (2012). IFN-α in the treatment of melanoma. J. Immunol..

[215] Kowalzick L., Rogozinski T., Wimheuer R., Pilz J., Manske U., Scholz A., Fierlbeck G., Mohr P., Ochsendorf F., Wagner G. (2002). Intralesional recombinant interferon beta-1a in the treatment of basal cell carcinoma: Results of an open-label multicentre study. Eur. J. Dermatol..

[216] Turan A., Saricaoglu H., Baskan E.B., Toker S.C., Tunali S. (2009). Treatment of infiltrating basal cell carcinoma with the combination of intralesional IFNalpha-2b and topical imiquimod 5% cream. Int. J. Dermatol..

[217] Migden M.R., Rischin D., Schmults C.D., Guminski A., Hauschild A., Lewis K.D., Chung C.H., Hernandez-Aya L., Lim A.M., Chang A.L.S. (2018). PD-1 Blockade with Cemiplimab in Advanced Cutaneous Squamous-Cell Carcinoma. N. Engl. J. Med..

[218] Maubec E., Boubaya M., Petrow P., Beylot-Barry M., Basset-Seguin N., Deschamps L., Grob J.-J., Dréno B., Scheer-Senyarich I., Bloch-Queyrat C. (2020). Phase II Study of Pembrolizumab as First-Line, Single-Drug Therapy for Patients With Unresectable Cutaneous Squamous Cell Carcinomas. J. Clin. Oncol..

[219] Saha K., Hornyak T.J., Eckert R.L. (2013). Epigenetic cancer prevention mechanisms in skin cancer. AAPS J..

[220] Zhang P.F., Li Y.S., Wang C., Gao Y.H., Liu J.Y., Zhang H.E., Shi L., Sun L.P. (2026). Recent advances and strategies in BET bro-modomain inhibition for drug discovery. Eur. J. Med. Chem..

[221] Tigu A.B., Ivancuta A., Uhl A., Sabo A.C., Nistor M., Mureșan X.-M., Cenariu D., Timis T., Diculescu D., Gulei D. (2025). Epigenetic Therapies in Melanoma—Targeting DNA Methyla-tion and Histone Modification. Biomedicines.

[222] Liguori L., Luciano A., Pagliara V., Polcaro G., De Feo R., Viggiano A., Salomone F., Avanzo A., Vitale F., D’ambrosio S. (2025). Epigenetic modifiers to enhance the efficacy of immune checkpoint inhibitors for the treatment of melanoma. Transl. Oncol..

[223] Xiao X., Hu X., Yao J., Cao W., Zou Z., Wang L., Qin H., Zhong D., Li Y., Xue P. (2023). The role of short-chain fatty acids in inflammatory skin diseases. Front. Microbiol..

[224] Paciolla C., Manganelli M., Di Chiano M., Montenegro F., Gallone A., Sallustio F., Guida G. (2025). Valeric Acid: A Gut-Derived Metabolite as a Potential Epigenetic Modulator of Neuroinflammation in the Gut–Brain Axis. Cells.

